# A 7-Week Summer Camp in Antarctica Induces Fluctuations on Human Oral Microbiome, Pro-Inflammatory Markers and Metabolic Hormones Profile

**DOI:** 10.3390/microorganisms11020339

**Published:** 2023-01-30

**Authors:** Michele M. Moraes, Thiago T. Mendes, Leandro Borges, Alice L. Marques, Cristian Núñez-Espinosa, Dawit A. P. Gonçalves, Carolina B. Simões, Tales S. Vieira, Roberto V. P. Ladeira, Talita G. B. Lourenço, Danielle V. Ribeiro, Elaine Hatanaka, Debora Heller, Rosa M. E. Arantes

**Affiliations:** 1 Department of Pathology, Institute of Biological Sciences, Universidade Federal de Minas Gerais, Belo Horizonte 31270-901, MG, Brazil; 2 Center for Newborn Screening and Genetics Diagnosis, Faculty of Medicine, Universidade Federal de Minas Gerais, NUPAD-FM/UFMG, Belo Horizonte 30130-100, MG, Brazil; 3 Department of Physical Education, Faculty of Education, Universidade Federal da Bahia, Salvador 40170-110, BA, Brazil; 4 Interdisciplinary Program in Health Sciences, Universidade Cruzeiro do Sul, São Paulo 01506-000, SP, Brazil; 5 Post-Graduation Program in Social Sciences in Development, Culture and Society of the Universidade Federal Rural do Rio de Janeiro, Seropédica 23890-000, RJ, Brazil; 6 School of Medicine, Universidad de Magallanes, Punta Arenas 6200000, Chile; 7 Austral Integrative Neurophysiology Group, Centro Asistencial Docente y de Investigación, Universidad de Magallanes, Punta Arenas 6200000, Chile; 8 Interuniversity Center for Healthy Aging, Punta Arenas 6200000, Chile; 9 Exercise Physiology Laboratory, School of Physical Education, Physiotherapy and Occupational Therapy, Universidade Federal de Minas Gerais, Belo Horizonte 31270-901, MG, Brazil; 10 Sports Training Center, School of Physical Education, Physiotherapy and Occupational Therapy, Universidade Federal de Minas Gerais, Belo Horizonte 31270-901, MG, Brazil; 11 Oral Microbiology Laboratory, Institute of Microbiology Paulo de Góes, Universidade Federal do Rio de Janeiro, Rio de Janeiro 21941-902, RJ, Brazil; 12 Hospital Israelita Albert Einstein, São Paulo 05652-900, SP, Brazil; 13 Post-Graduate Studies in Dentistry, Universidade Cruzeiro do Sul, São Paulo 430-0926, SP, Brazil; 14 Department of Periodontology, School of Dentistry, UT Health San Antonio, San Antonio, TX 78229, USA

**Keywords:** catecholamines, cytokines, human microbiome phyla, inflammation, markers, oral microbiome, polar, stress-related responses

## Abstract

Antarctic camps pose psychophysiological challenges related to isolated, confined, and extreme (ICE) conditions, including meals composed of sealed food. ICE conditions can influence the microbiome and inflammatory responses. Seven expeditioners took part in a 7-week Antarctic summer camp (Nelson Island) and were evaluated at Pre-Camp (i.e., at the beginning of the ship travel), Camp-Initial (i.e., 4th and 5th day in camp), Camp-Middle (i.e., 19th–20th, and 33rd–34th days), Camp-Final (i.e., 45th–46th day), and at the Post-Camp (on the ship). At the Pre-Camp, Camp-Initial, and Camp-Final, we assessed microbiome and inflammatory markers. Catecholamines were accessed Pre- and Post-Camp. Heart rate variability (HRV), leptin, thyroid stimulating hormone (TSH), and thyroxine (T4) were accessed at all time points. Students’ *t*-tests or repeated-measures analysis of variance (one or two-way ANOVA) followed by Student-Newman-Keuls (*post hoc)* were used for parametric analysis. Kruskal-Wallis test was applied for non-parametric analysis. Microbiome analysis showed a predominance of *Pseudomonadota* (34.01%), *Bacillota* (29.82%), and *Bacteroidota* (18.54%), followed by *Actinomycetota* (5.85%), and *Fusobacteria* (5.74%). Staying in a long-term Antarctic camp resulted in microbiome fluctuations with a reduction in *Pseudomonadota*—a “microbial signature” of disease. However, the pro-inflammatory marker leptin and IL-8 tended to increase, and the angiogenic factor VEGF was reduced during camp. These results suggest that distinct Antarctic natural environments and behavioral factors modulate oral microbiome and inflammation.

## 1. Introduction

Researchers have annually traveled to Antarctica to carry out their scientific activities on ships and in research stations and camps. Camps pose a particular environmental challenge due to extensive cold exposure, extreme lighting conditions, physical effort during outdoor fieldwork, storms, and white-out situations that are summarized by the ICE acronym (Isolation, Cold and Extreme conditions). Some of these conditions have been investigated and influence psychophysiological parameters [1,2,3,4]. Staying in camps also implies purchasing in advance and transporting all the provisions to consume in the expedition; resulting in a low supply of fresh and in natura foods; thus, meals would be expected to be mainly composed of processed and ultra-processed foods [5,6].

The human microbiome, the ecological community of microorganisms that share our body space [7], is determined by microbiome-intrinsic and -extrinsic factors. Schmidt et al. [8] present microbiome extrinsic factors as (i) host-intrinsic: including immunity and body index mass; (ii) host-extrinsic, i.e., lifestyle (including physical activity and diet); and (iii) environmental [8]. Therefore, since factors such as environment and latitude/geographical localization [9,10,11,12], food and nutrient intakes [9,13,14], stressors [15,16], physical exercise [17], and body temperature [18] influence the microbiome, it is plausible that permanence in ICE modulates the microbiome—which can indirectly impact host health [8,9].

Microbial populations reside on numerous sites of the body; although analyses are usually conducted in feces, these measurements can also be performed in saliva samples [7,19,20]. The oral cavity has the second largest and most diverse microbiota in the human body [20], with the microbiome representing approximately 30–60% of total saliva DNA [19,21]. Salivary measures are a non-invasive source of biomarkers and present advantages to the facility of collection and storage and the acceptance of volunteers [22]—methodological characteristics required during an expedition in an inhospitable local such as Antarctica.

The composition of the oral microbiome modulates cytokine production and reflects oral and overall health, once changes in the oral ecosystem can result in caries, gingivitis, and periodontitis [7,23]. Sarkar et al. [19] have shown that both oral inflammations, as evaluated by salivary interleukins (IL’s) IL-6, IL-8, and IL-1β, and salivary microbiome present a similar oscillatory pattern. Importantly, environmental factors such as prolonged exposure to natural sunlight and ultraviolet radiation [24] and cold [25] may influence cytokine responses due to stressful elements and, therefore, may influence the systemic immune system. Also, proinflammatory cytokines can induce vascular endothelial grow factor (VEGF), a proangiogenic protein.

Additionally, the stressful elements present in the ICE environment can induce sympathetic hyperactivity, changing cardiac autonomic tone and increasing in the concentration of circulating catecholamines. The stressful elements and thermal discomfort caused by cold temperatures increase metabolic demand, thus stimulating endocrine responses that trigger thermogenesis, such as releasing thyroid hormones [26]. It is worth noting that individuals with higher levels of thyroid-stimulating hormone (TSH) present a higher microbiome diversity [27], which is associated with indicators of physical health [28].

Thus, this study aimed to assess the effects of a 7-week camp during an Antarctic summer (December–January) on the microbiome, systemic inflammatory markers, sympathetic/parasympathetic autonomic balance (heart rate variability, HRV), and stress-related hormones (catecholamines and thyroid hormones). Also, the dietary pattern in the camp was characterized.

## 2. Materials and Methods

*Ethics*

This study followed the regulations established by the Brazilian National Health Council (resolution 466/2012), and the research protocol was approved by the Research Ethics Committee of the Universidade Federal de Minas Gerais (19092819.8.0000.5149/3.744.162). The volunteers were informed about the research objectives and all the experimental procedures before giving their written informed consent for participation in this study.

*Subjects and experimental approach*

Considering the study’s characteristics—accompanying a group of expeditionaries during a camp in Antarctica, all individuals in the group were invited to participate in the study. Seven volunteers (five men and two women) [age 32.3 ± 8.4 yr., body mass index (BMI) 23.1 ± 4.5 kg/m^2^] were recruited and accepted to participate in this study, which followed a descriptive longitudinal approach. To carry out the expedition, all participants presented a certificate issued by a medical doctor to prove their physical health (according to the model presented in Supplemental Material S1) and a certificate issued by a dentist, including declarations that they did not have (i) inflammatory processes and/or acute or chronic infections of the oral cavity or periodontal diseases; (ii) soft or hard tissue tumor lesions; (iii) untreated or insufficiently treated carious processes; (iii) an insufficient number of natural or artificial dental elements that do not ensure satisfactory chewing; (iv) any incomplete dental treatment.

The entire expedition lasted 62 days: seven days on board Brazil’s Navy polar ship “Ary Rongel”, 50 days living in a camp settled in Nelson Island located in the South Shetland Islands (S 53.178533°/O 70.899750°), and five days on Brazil’s Navy polar ship “Almirante Maximiano” (number of tack H-41). Our experiment was conducted between December 2019 and February 2020, during the Antarctic summer season.

**Figure 1:**
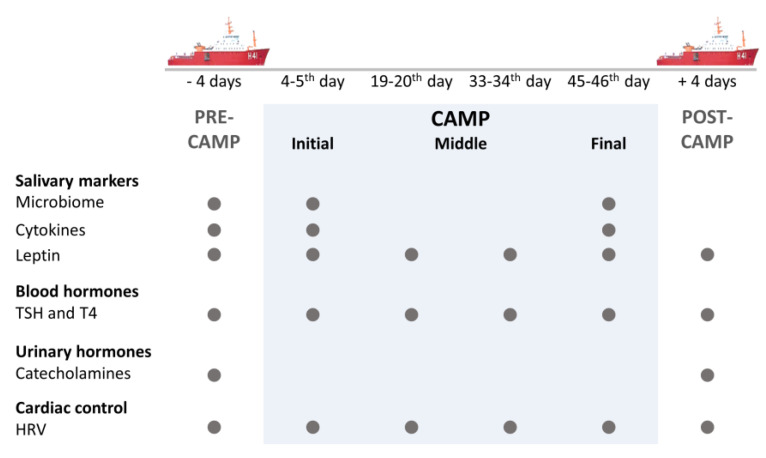
Timeline of data collection. The expedition lasted 62 days: seven days on ship, 50 days (49 nights) in camp, and five days on the polar ship. Data were collected at the following moments: Pre-Camp (i.e., at the beginning of the ship travel, at 2nd and 3rd days on the ship), at camp in the moments: Camp-Initial (i.e., 4th and 5th day in camp), Camp-Middle (i.e., 19th–20th, and 33rd–34th days in camp), and Camp-Final (i.e., 45th–46th day in camp), and at the Post-Camp, (i.e., in the final of the ship travel, at 4th day on the ship). The gray circles represent the measurements made at each time point. Abbreviations: thyroid-stimulating hormone (TSH), thyroxine (T4), heart rate variability (HRV).

Data collection was carried out Pre-Camp (i.e., at the beginning of the ship travel, on 2nd and 3rd days on the ship), at camp in the moments: Camp-Initial (i.e., 4th and 5th day in camp), Camp-Middle (i.e., 19th–20th, and 33rd–34th days in camp), and Camp-Final (i.e., 45th–46th day in camp), and at the Post-Camp (i.e., in the final of the ship travel, at 4th day on the ship) (Figure 1). The Pre-Camp data collection was carried out on the ship in the Antarctic maritime, as the crossing from Chile to Antarctica was carried out via a flight to the Chilean Base Presidente Eduardo Frei Montalva. Regarding the field expedition, the first data collection (Camp-Initial) was performed on the 4th day (for salivary and questionnaires) and 5th day (for blood and cardiovascular autonomic control) because on the first three days, the group assembled the camping structures. After the removal of the camping equipment and boarding of the group on the ship, Post-Camp data collection was carried out following the cross of Drake’s Passage. During data collection on the ship and at camp, the volunteers followed the same experimental protocol.

The microbiome and inflammatory markers (IL1-β, IL-6, IL-8, Tumor Necrosis Factor-α human-TNF-α, high-sensitivity C-reactive protein-hs-CRP, and VEGF) were accessed at the Pre-Camp, Camp-Initial, and Camp-Final. The catecholamines were evaluated in urine collected Pre- and Post-Camp. HRV, leptin, TSH, and thyroxin (T4) were accessed at all time points. The anthropometric characteristics of the participants were recorded at the Camp-Initial and Camp-Final (Table 1). In addition, the dietary pattern was registered along the camp (from Camp-Middle to Camp-Final) to characterize the food intake.

During camp, individuals kept a regular diurnal schedule with field trips throughout the working days. The average walking distance during fieldwork was 4.1 ± 0.8 km [0.2 (min) to 16.7 km (max)] per day. During the field period, there was long days’ length (from 19:42:19 to 17:24:22, https://www.timeanddate.com/sun/@6622142?month=1&year=2020, accessed on 14 December 2022) and a few hours of twilight.

*Procedures*

*Assessment of anthropometric characteristics*

Body mass was measured with volunteers wearing shorts (men) or shorts and a top (women). Skinfold thickness was measured at seven different sites (triceps, subscapular, pectoral, mid-axilla, abdominal, supra iliac, and mid-thigh); skinfolds were measured by the same individual to the nearest millimeter in duplicate using a skinfold caliper (Lange, MI, USA) and the average value was recorded. These seven measures were summed to determine the ∑skinfolds. Body fat was calculated according to the protocol proposed by Jackson and Pollock [29].

*Salivary samples and analyses*

A 2.0-mL unstimulated whole saliva (uWS) was obtained for the determination of salivary microbiome concentrations, as in previous studies [30]. Also, once when using passive drool methods, it is recommended to collect at least 200 μL for each assay. A 1.5-mL uWS sample was obtained to determine the concentration of salivary immunological and inflammatory markers (for seven markers) [31]. In addition, a separate 0.5-mL uWS sample was obtained for the determination of salivary cortisol. The uWS samples were collected in the Antarctic field, stored in liquid nitrogen during the camp period, and, after the days in the camp, transferred to the −80 °C freezer inside the ship. The samples were kept frozen (−80 °C) until the moment of processing and analysis. The tubes containing saliva samples were centrifuged (Eppendorf, 5430 R) at 14,000× *g* for 20 min at 4 °C, next the supernatant and the pellet were aliquoted. Microbiome analyzes were performed on the whole saliva pellet. Analyzes of immunological and hormonal markers were performed on the whole saliva supernatant.

*Microbiome analysis*

*Library Preparation and 16S Sequencing*

Genomic DNAs were extracted using PowerSoil DNA Isolation Kit (MO BIO Laboratories, Inc. Carlsbad, CA, USA) according to the manufacturer’s instructions, and the 16S rRNA V3 and V4 variable regions (341F-CCTACGGGNGGCWGCAG and 805R-GACTACHVGGGTATCTAATCC) were amplified using the kit QIAseq 16S/ITS Region Panels (Qiagen, Quiagen N.V, Venlo, Netherlands). Before the PCR reaction, all the DNA samples were quantified by Qubit Fluorometer using Qubit™ dsDNA HS Assay Kit (Thermo Fisher Scientific, Waltham, MA, USA) and diluted to 1 ng/uL.

PCR reaction was carried out with 5.0 μL of 1ng/uL Microbial DNA sample, 2.5 μL UPC Master Mix, 5.5 μL UPC PCR Water for a final reaction volume of 10 µL and to set up the PCR reactions: 95 °C for 5 min, followed by 12 cycles of 95 °C for 30 s, 50 °C for 30 s, 72 °C for 2 min, and an extension of 72 °C for 7 min. The purification was performed with 40 uL de UPC PCR water for reaction and 55 uL de QIAseq beads, followed by two rounds of purification with 200 uL of 80% ethanol and dilution of the purified amplicon in a total of 35 uL UPC PCR Water.

Adapters were added to the ends of amplicons, and libraries were generated using the QIAseq 16S/ITS index kit (Qiagen): 12.5 uL UPC Master Mix, 5 uL UPC Water, and 32.5 uL amplicon, a total of 50 uL were added to the index plate. The PCR conditions for the index PCR reaction were as follows: 95 °C for 2 min, followed by 19 cycles of 95 °C for 30 s, 60 °C for 30 s, 72 °C for 2 min, and an extension of 72 °C for 7 min; and then they were purified as previously described.

The information of the appropriate fragment size for each sample was performed on Agilent 2200 TapeStation system, and the quantification of the libraries was performed by Qubit Fluorometer using Qubit™ dsDNA HS Assay Kit (Thermo Fisher Scientific, Waltham, MA, USA) according to the manufacturer’s instructions.

Libraries were diluted to 2 nM based on the Qiaseq Library Quant Assay Kit (Qiagen) measurements and the size information performed on Agilent 2200 TapeStation system. For pool preparation, 5 uL of each 2 nM library were joined. For pool denaturation, 5 µL of the 2 nM pool was added with 5 µL of 0.2 N NaOH, and after the denaturation, 990 µL of Illumina’s HT1 buffer was added to the pool to dilute it to 10 pM. The diluted was spiked with 1% PhiX and sequenced using a MiSeq Reagent Kit v3 (600-cycle) (Illumina, San Diego, CA, USA) [32].

*Bioinformatics data analysis*

Bioinformatics analyses were performed using Mothur software v. 1.44.3 (https://www.mothur.org, accessed on 14 December 2022) [33]. Forward and reverse paired sequences were grouped into contigs, and their barcodes and primers were removed from sequences. Sequences containing ambiguities (N-base) or containing more than 8-mer homopolymers were removed. All sequences presenting inconsistent sizes from expected for the amplicon were also removed. Unique sequences were grouped through the “unique.seqs” command. A virtual PCR was done in the Silva Ribosomal database v.138.1 [34] using the 341F and 805R primers to train the dataset. Once this step has been performed, a new virtual PCR was done with HOMD database, and the sequences were then aligned with HOMD database [35]. Badly aligned sequences and non-informative columns were eliminated. All sequences were trimmed to fully overlap, and unique sequences were again grouped.

Pre-clustering of the sequences with a different threshold of 2 bp was done. The chimeras were checked and removed using the “chimera.vsearch” command. The resulting reference file was used to classify the sequences using an 80% bootstrap threshold. Sequences from mitochondria, chloroplasts, Eukarya, Archaea, and unknown domains were removed. OTU clustering was performed with a 3% similarity cutoff, and singletons were removed. All samples were normalized based on the size of the smallest one (75.255 sequences) by random subsampling. Rarefaction curves, alpha diversity indexes, the relative abundance of taxa, and an OTU distribution matrix were exported from the software. After the quality filter steps, one sample was removed because of the low number of sequences. Raw sequence data were deposited in the NCBI Sequence Read Archive (SRA) and are available under Bio project accession number PRJNA695574. The segment of 283pb corresponding to the V3–V4 region of the 16S rRNA gene from saliva samples of all volunteers generated 2.213.811 sequences, with a total number of 345.210 assigned OTUs.

The community diversity was calculated using the Shannon index (more sensitive to species richness) [36] and Simpson’s Index (more sensitive to species evenness) [36], while total species richness was calculated using the Chao-1 index [37].

*Salivary analyses of the inflammatory biomarkers and leptin*

Before salivary analysis, the mucins and precipitants were removed by centrifugation of the samples. Cytokines [IL1-β, IL-6, IL-8], TNF-α, hs-CRP, VEGF, and leptin were measured according to the manufacturer’s protocols using enzyme-linked immunosorbent assay (ELISA) (DuoSet Kit; Quantikine, R&D Systems, Minneapolis, MN, USA) (dilution 1:3 for IL1-β and hs-CRP, 1:1 for TNF-α and VEGF, and no dilution for IL-8, IL-6, and leptin). Once the dilution of saliva samples can improve the detectability of inflammatory markers [31,38,39] when necessary, adequate dilutions were tested, and the one with the best detectability was used. The optical density (OD) of each well was immediately assessed with a microplate reader set to 450 and 540 nm (wavelength correction). The final values were normalized to the total salivary protein (Pierce™ BCA Protein Assay Kit, ThermoFisher Scientific, Waltham, MA, USA). Ten percent of the samples were assessed in duplicate, and the intra-assay coefficient of variance was 3.9% for IL1-β, 7.0% for IL-8, 15.9% for TNF-α, 10.4% for hs-CRP, 6.6% for VEGF, 10.1% for IL-6, and 12.6% for leptin. The data were linearized by plotting the log of the cytokine levels vs. the log of the OD, and the fitting line was defined by regression analysis. The IL-6 amplitude was calculated as the difference between the 7:00 am and 7:00 pm concentrations.

*Blood samples and thyroid hormones measures*

For measuring thyroid hormone concentrations, drops of blood were collected on a filter paper (Whatman S&S 903 Screening Cards, Life Sciences, GE Healthcare, Chicago, IL, USA), dried horizontally, stored in plastic bags with silica, and kept away from exposure to light and hot temperatures [40]. Dried blood spot disks (each 3.2 mm in diameter) were plated, diluted in europium buffer solution, and analyzed by time-resolved two-site fluoroimmunoassay with direct double-sandwich technique (GSP Neonatal hTSH and GSP Neonatal T4; WallacOy, Turku, Finland), as previously reported in a similar population [1,41,42], CV: TSH = 8.3% and T4 = 7.6%.

*Urinary samples and catecholamines measures*

The urine was collected during a 12 h period (overnight) and mixed before reaching a 10-mL sample, stored acidified, and homogenized with 50% hydrochloric acid (pH 3 to 4). After identification, the urine samples were stored at −80 °C until analysis. The catecholamine concentration was measured by liquid chromatography coupled to mass spectrometry (in-house Method) (Quattro Micro, Waters Corporation, MA, USA) [43], CV: 6%.

*HRV measures for cardiac autonomic control analyses*

For heart rate (HR) measurements, a moistened elastic electrode strap with a Polar H10 cardiac transducer (Polar Electro Oy, Kempele, Finland) was fitted below the volunteer chest muscles, and the strap length was fitted to chest circumference. HRV was determined via recordings of RR intervals (intervals between successive R-waves) (Polar^®^ S810i; Polar, Finland), with the volunteers remaining seated on a chair for 20 min inside the ship and in the tent at camp. Data analysis was performed as described previously [44]. Briefly, the RR intervals were continuously recorded during the last 10 min of rest, and 5 min extracts were analyzed. Kubios HRV^®^ free software (University of Eastern Finland) was used to analyze the data. The time-domain parameters—square root of the mean squared differences of successive RR intervals (RMSSD) expressed in milliseconds (ms), the natural log of RMSSD (Ln(RMSSD)), the number of interval differences of successive normal-to-normal (NN) intervals greater than 50 ms (NN50), and the percentage of adjacent RR intervals with a time difference greater than 50 ms (pNN50)—were calculated.

The spectral power was expressed in ms^2^ and integrated into the high-frequency (HF; 0.15 to 0.40 Hz) and low-frequency (LF; 0.04 to 0.15 Hz) power bands. The HF power band reflects the parasympathetic influence and is related to respiratory sinus arrhythmia [45], whereas the LF power band and the LF/HF ratio have been associated with baroreflex activity [46].

*Dietary pattern assessment (Food recall questionnaire)*

The 24 h food recall questionnaire (FRQ) was applied in personal interviews to capture detailed information about foods and beverages consumed. FRQ was completed by each volunteer at night before going to sleep in his/her individual tent. On the morning of the following day, the researcher asked the volunteer about any additional food that could have been consumed after completing FRQ the previous night. The amount of each type of food consumed was estimated according to a standard measuring kitchen utensil available in the field (e.g., spoon, cup, etc.). Data extracted from FRQ were analyzed with DietBox^®^ software (version 8.0.9, Porto Alegre, Brazil). Food consumption was classified and calculated for processing level in accordance with the NOVA classification [5,6]: in natura and minimally processed foods, as processed and ultra-processed foods (Supplemental Material S2).

*Statistical analyses*

Normality of data was tested using Shapiro-Wilk test. The Shapiro–Wilk test revealed that all the parameters evaluated did not show a significant departure from the normal distribution, except T4 and, for microbiome at phylum level, *Spirochaetes*, submitted to log-transform, *Gracilibacteria*, submitted to reciprocal-transform, and *Synergistetes* submitted to rank-transform; these data were then analyzed as normally distributed data. At the genus level, non-normal data were transformed; however, for data that did not meet the criteria for parametric analysis, non-parametric analysis (Kruskal–Wallis test) was used. The equal variance was tested and confirmed using the Levene median test. Test details of data of the normality and the equivariance at phylum and genus level are presented in Supplemental Material S3. Data are shown as mean ± standard deviation (SD). Grubbs’ test was applied to identify outliers [47].

One-way repeated-measures analyses of variance (ANOVA) were used to compare microbiome (phyla and genus) and indexes, salivary cytokines, and signal proteins (IL1-β, IL-8, IL-6, hs-*CRP,* TNF-α, and VEGF), leptin, TSH and T4, and HRV measures across time points during the expedition. When a significant *F* value was found in One-way and two-way ANOVA tests, respectively, Student-Newman-Keuls was performed as *post hoc* analyses. Paired students’ *t*-tests were used to compare two means. The *α* level was set at 0.05. Spearman’s correlation (for comparisons involving microbiome data) or Pearson’s correlation (for other comparisons) were used to evaluate the strength of a linear association between two variables by determining the *r* coefficient. The *r* coefficient effect size values were classified as small (0.2–0.5 or −0.2–−0.5), medium (0.5–0.8 or −0.5–−0.8), and large (>0.8 or <−0.8) [48]. Descriptive statistics were performed using the Excel (Microsoft Corporation, 2018) and inferential statistics analyses were performed using the SigmaPlot 11.0 software (Systat Software Inc., San Jose, CA, USA). The α level was set at 0.05.

Considering the limited number of subjects joining the expedition, we also calculated Cohen’s d effect size (*ES*) as a supplementary analysis. Cohen’s d was calculated by subtracting the mean value for one group from the mean value of the other group. The result was then divided by a combined SD of the data. The *ES* for ANOVAs was calculated using the following equation *η*2 = Effect SQ/Total SQ; SQ = sum of squares. The *η*2 values were converted into *d* values [49]. The ES values were classified as trivial (*ES* < 0.2), small (*ES* 0.2–0.6), medium (*ES* 0.6–1.2), or large (*ES* ≥ 1.2) [50].

## 3. Results

### 3.1. The Transition to Antarctic Camp and Stay in the Field Resulted in Fluctuation in the Microbiome

**Figure 2:**
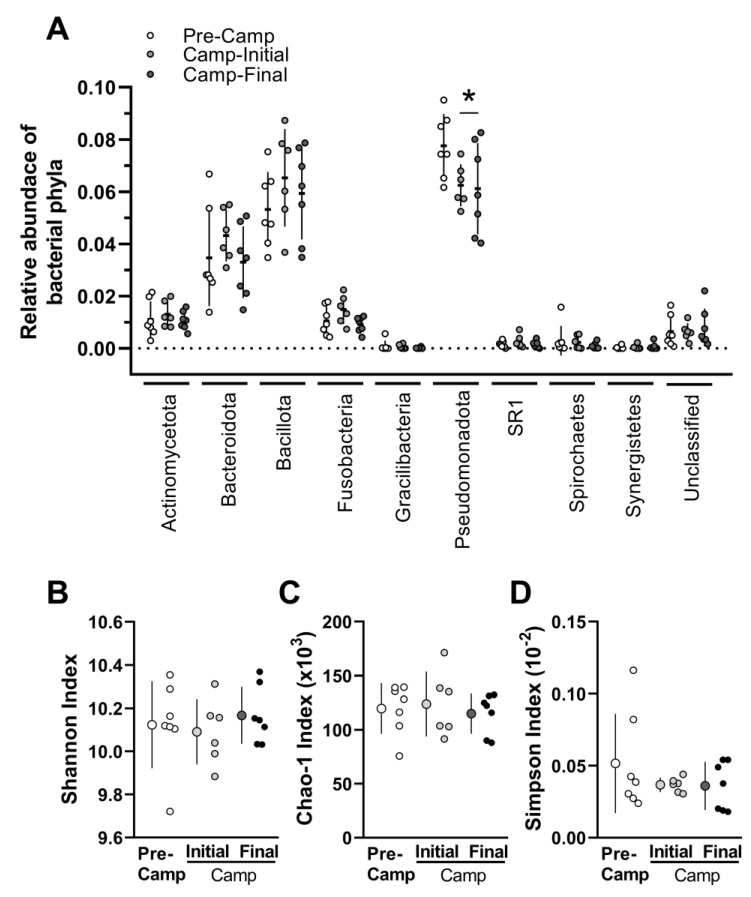
Microbiome abundance and diversity data the Pre-Camp (i.e., 2nd and 3rd days on the ship, before camp; white circles), and at Camp [Initial (i.e., 4th day in camp; grey circles), and Final (i.e., 45th day in camp; black circles) moments at camp] during an Antarctic expedition. (**A**) Relative abundance of bacterial phyla, considering all OTU—the identified and unidentified in some phylum/genus/species (unknown). (**B**) Shannon Index. (**C**) Chao-1 Index. (**D**) Simpson Index. n = 7, except for the Camp-Initial, n = 6, due to low DNA concentration for one volunteer. *Pseudomonadota*, former *Proteobacteria*. *Bacillota*, former *Firmicutes*. *Bacteroidota,* former *Bacteroidetes*. *Actinomycetota*, former *Actinobacteria*. The data are expressed as mean ± SD. The dots represent the individual datum. * Significantly different (*p* < 0.05) from the Pre-Camp.

Microbiome analysis showed nine different phyla, with a predominance of *Pseudomonadota* (former *Proteobacteria*), *Bacillota* (former *Firmicutes*), and *Bacteroidota* (former *Bacteroidetes*), followed by *Actinomycetota* (former *Actinobacteria*), and *Fusobacteria* (Figure 2A; for individual data see Supplemental Material S4, Figure S4), with a total of 104 genera detected in at least one sample (microbiome species detected are presented in Supplemental Material S4, Table S4). A 7-week camp led to a large reduction of *Pseudomonadota* (*F* = 4.280; *p* = 0.042; *ES* = 1.3), with no differences for the other phyla or Shannon, Chao-1 and Simpson Indexes (Figure 2B–D) [*Actinomycetota*: *F* = 1.491, *p* = 0.267; *ES* = 0.6; *Bacteroidota F* = 0.586, *p* = 0.573; *ES* = 0.4; *Bacillota*: *F* = 0.915, *p* = 0.429; *ES* = 0.6; *Fusobacteria*: *F* = 2.436, *p* = 0.133; *ES* = 0.8; *Gracilibacteria*: *F* = 0.836, *p* = 0.459; *ES* = 0.5; SR1: *F* = 1.216, *p* = 0.333; *ES* = 0.5; *Spirochaetes*: *F* = 0.672, *p* = 0.530; *ES* = 0.2; *Synergistete*: *F* = 0.551, *p* = 0.591; *ES* = 0.2; unclassified: *F* = 0.852, *p* = 0.453; *ES* = 0.4; Shannon Index: *F* = 0.512, *p* = 0.613; *ES* = 0.4; Chao-1 Index: *F* = 0.163, *p* = 0.852; *ES* = 0.1; Simpson Index: *F* = 1.942, *p* = 0.190; *ES* = 0.7]. The ANOVA of the isolated *Pseudomonadota* genera showed a reduction in *Pasteurellaceae* (unclassified) (*F* = 6.584, *p* = 0.013; *ES* = 1.6) and a tendency towards a reduction in *Neisseria* (*F* = 3.128, *p* = 0.084; *ES* = 1.0), with no differences for other individual genera analyzed.

The dispersion analysis by the coefficient of variation (CV) showed changes along with the situations (*F* = 3.843, *p* = 0.041; *ES* = 0.4), with a reduction in the CV between Pre-Camp and Camp-Initial (*p* = 0.033), with no differences between Camp-Initial and Camp-Final (*p* = 0.242).

**Figure 3:**
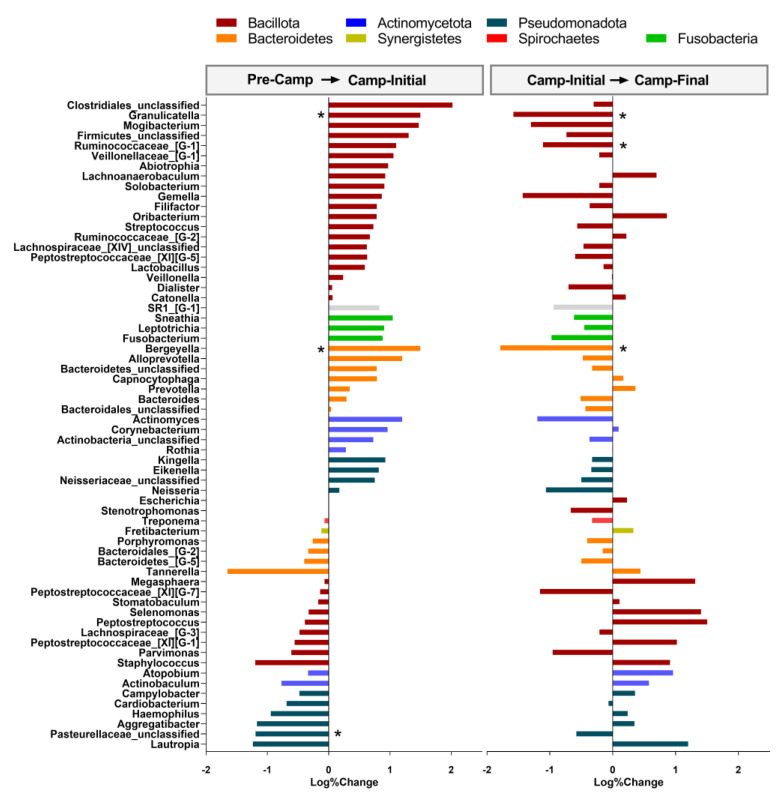
Changes in the relative abundance of bacterial genus of the microbiome. Changes between Pre-Camp and Camp-Initial (i.e., Camp-Initial-Pre-Camp; **left panel**) and between Camp-Initial and Camp-Final (i.e., Camp-Final-Camp-Initial; **right panel**). Colors represent different phyla. Data presented as the logarithm of percentual of change in relative abundance. n = 7, except for the Camp-Initial, n = 6, due to low DNA concentration for one volunteer. *Pseudomonadota*, former *Proteobacteria*. *Bacillota*, former *Firmicutes*. *Bacteroidota,* former *Bacteroidetes*. *Actinomycetota*, former *Actinobacteria*. * Significantly different (*p* < 0.05) from the Pre-Camp (for the **left panel**) or the Camp-Initial (for the **right panel**).

Changes were also observed at the genus level. ANOVA showed differences for *Granulicatella* (*F* = 7.276, *p* = 0.010; *ES* = 1.4), *Bergeyella* (*F* = 4.078, *p* = 0.047; *ES* = 1.2)*, Ruminococcaceae*-[G-1] (*F* = 8.057, *p* = 0.007; *ES* = 1.0), *Pasteurellaceae*-*unclassified* (*F* = 4.869, *p* = 0.031; *ES* = 1.4). Specifically, *post-hoc* tests indicated that the transition from ship to camp (i.e., comparison Pre-Camp vs. Camp-Initial) increased *Granulicatella (p* = 0.01), *Bergeyella (p* = 0.04), and a decrease in *Pasteurellaceae*-*unclassified (p* = 0.05). Compared to Camp-Initial, Camp-Final showed a reduction for *Granulicatella (p* = 0.01), *Ruminococcaceae*-[G-1] *(p* = 0.006), and *Bergeyella (p* = 0.05) (Figure 3). Also, there was a tendency to difference for *Clodtridiales-unclassified (F* = 3.830, *p* = 0.055; *ES* = 0.9), *Gemella (F* = 3.247, *p* = 0.078; *ES* = 1.1), and *Neisseria (F* = 3.128, *p* = 0.084; *ES* = 1.0). Comparisons of Camp-Final to Pre-Camp to assess the possible summation effect of changes throughout the whole period of our study showed a reduction in *Pasteurellaceae*-unclassified *(p* = 0.031).

### 3.2. Inflammatory Biomarkers and Leptin Salivary Analyses

**Figure 4:**
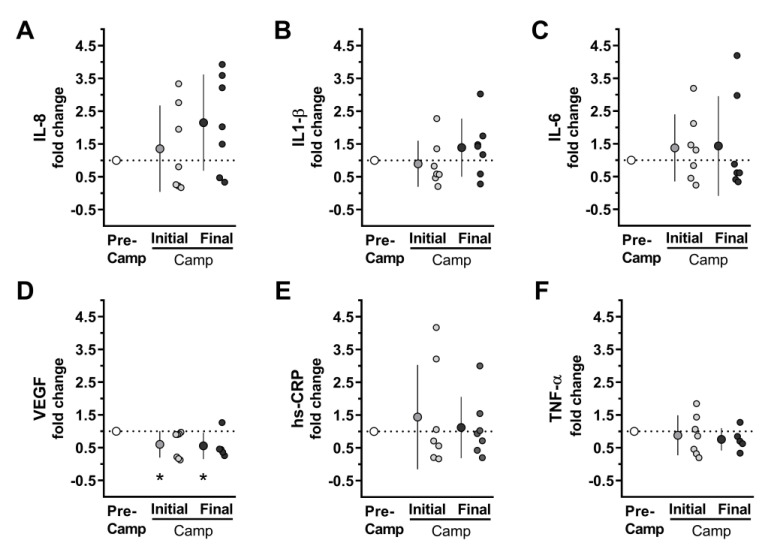
Salivary cytokine and signal proteins at the Pre-Camp (i.e., 2nd and 3rd days on the ship, before camp; white circles), and at Camp [Initial (i.e., 4th day in camp; grey circles), and Final (i.e., 45th day in camp; black circles) moments at camp] during an Antarctic expedition. (**A**) Interleukin-8 (IL-8). (**B**). Interleukin 1-beta (IL1-β) (**C**) Interleukin-6 (IL-6). (**D**) Vascular endothelial growth factor (VEGF). (**E**) High-sensitivity C-reactive protein *(hs-CRP)*. (**F**) Tumor necrosis factor-alpha (TNF-α). n = 7, except for TNF-*α* and VEGF at the Final moment, n = 5, due to the limited volume of samples for one volunteer and one outlier. The Camp-Initial and Camp-Final data were normalized to the Pre-Camp values. The normalization aimed to rescale the data for better visualization of the changes in the data set, considering the high variability between individuals [mean CV (coefficient of variation) of the variables: 0.50 for VEGF to 1.46 for IL-6]. The data are expressed as mean ± SD. The dots represent the individual datum. * Significantly different (*p* < 0.05) from the Pre-Camp.

The evaluation of inflammatory biomarkers in Camp-Initial and Camp-Final compared to Pre-Camp revealed a moderate effect and tendency for an increase in IL-8 (*F* = 2.86; *p* = 0.097; *ES* = 0.9) (Figure 4A). In contrast, there was a reduction with a large effect for VEGF (*F* = 9.264; *p* = 0.005; *ES* = 1.4) (Figure 4D). Figure 4 shows no changes for IL1-β (*F* = 1.314; *p =* 0.30; *ES* = 0.7), IL-6 (*F* = 0.542, *p* = 0.595; *ES* = 0.4), hs-CRP (*F* = 0.360; *p* = 0.70; *ES* = 0.1), and TNF-α (*F* = 0.236; *p* = 0.79; *ES* = 0.3) (Figure 4B,C,E,F). The analysis of circadian changes in IL-6 in Post-Camp moment shows no significant difference in both morning and evening (Table 2). Although ANOVA did not indicate a difference in leptin levels at any studied time, there was a moderate effect over time (*F* = 1.425; *p* = 0.24; *ES* = 0.7).

**Figure 5:**
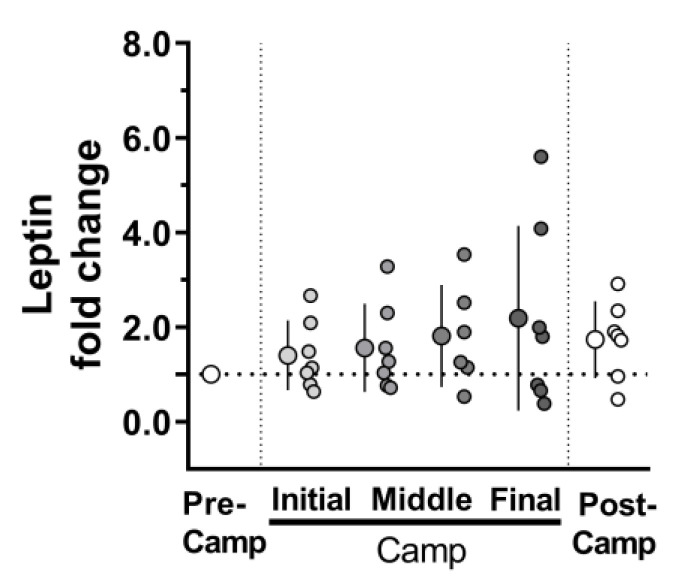
Salivary leptin at the Pre-Camp (i.e., 2nd and 3rd days on the ship, before camp; white circles), at Camp [Initial (i.e., 4th day in camp), in the Middle (19th and 33rd days in camp), and at Final (i.e., 45th day in camp) moments at camp; grey circles], and at the Post-Camp (i.e., 4th day on the ship, after camp; white circles) during an Antarctic expedition. n = 7. Data were normalized to the Pre-Camp values. The data are expressed as mean ± SD. The dots represent the individual datum.

A comparison between the two moments revealed a trend towards an increase in leptin in the Post-Camp compared to Pre-Camp (*F* = 5.670; *p* = 0.055; *ES* = 1.3). In addition, most of the volunteers presented higher leptin values at final of the expedition compared to Pre-Camp (Figure 5).

**Figure 6:**
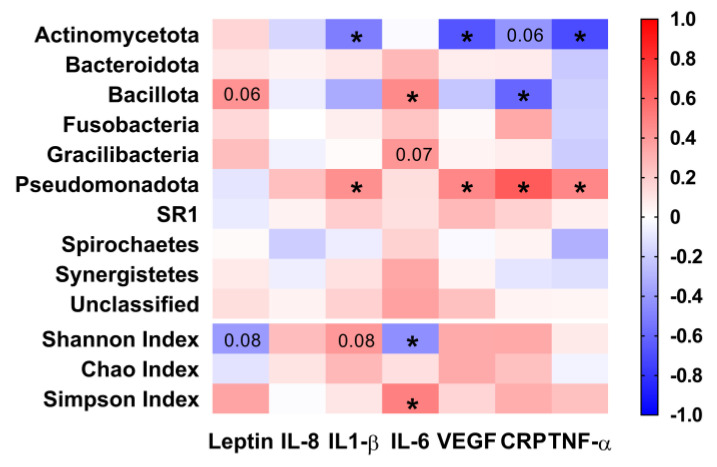
Heat map showing the magnitude of association between the relative abundance of bacterial phyla and diversity indexes with cytokine and signal proteins. IL-8: Interleukin-8. IL1-β: Interleukin 1-Beta. IL-6: Interleukin-6. VEGF: Vascular endothelial growth factor. hs-CRP: high-sensitivity C-reactive protein. TNF-*α*: Tumor necrosis *factor*-alpha. *Pseudomonadota*, former *Proteobacteria*. *Bacillota*, former *Firmicutes*. *Bacteroidota,* former *Bacteroidetes*. *Actinomycetota*, former *Actinobacteria*. Number of samples = 20 for all correlations, except for correlations with TNF-α and VEGF with a number of samples = 19, due to the limited volume of samples for one volunteer at the Final moment. * Significant association (*p* < 0.05) between the data.

Microbiome phyla presented an association with adipokines and cytokines (Figure 6), with moderate correlations (r = 0.50–0.80) of IL1-β, VEGF, and TNF-α with *Actinomycetota* (r = −0.51, *p* = 0.02; r = −0.67, *p* = 0.001; r = −0.71, *p* < 0.001, respectively), and hs-CRP with *Bacillota* (r = −0.60, *p* = 0.005) and with *Pseudomonadota* (r = 0.64, *p* = 0.002). It is also worth highlighting the correlations of IL-6 with *Bacillota* (r = 0.46, *p* = 0.04), Shannon Index (r = −0.44, *p* = 0.05) and Simpson Index (r = 0.50, *p* = 0.02), and IL1-β, VEGF and TNF-α with *Pseudomonadota* (r = 0.43, *p* = 0.05; r = 0.47, *p* = 0.04; r = 0.47, *p* = 0.04, respectively). In addition, there was a tendency to a correlation of leptin with *Bacillota* (r = 0.42, *p* = 0.06), leptin and IL1-β with Shannon Index (r = −0.39, *p* = 0.08; r = 0.40, *p* = 0.08, respectively), IL-6 with *Gracilibacteria* (r = 0.41, *p* = 0.07), and hs-CRP with *Actinomycetota* (r = −0.42, *p* = 0.06).

At the genus level, we highlight the moderate correlations of IL-8 with *Sneathia* and *Bergeyella*, IL1-β with *Granulicatella*, *Sneathia*, *Bacteroidales*-*unclassified*, and *Stenotrophomonas*, VEGF with *Granulicatella*, and *Stenotrophomonas*, hs-CRP with *Ruminococcaceae*-[G-2], *Rothia*, and *Stenotrophomonas*, and TNF-α with *Mogibacterium*, *Bacteroidales*-*unclassified*, *Rothia*, and *Stenotrophomonas* (for viewing the correlation coefficients of all associations, please see Supplemental Material S5).

There were positive correlations between leptin and IL-6 (r = 0.847, *p* < 0.001; 21 points), IL-8 and IL1-β (r = 0.703, *p* < 0.001; 21 points), VEGF and IL1-β (r = 0.49, *p* = 0.03; 20 points), VEGF and IL-8 (r = 0.51, *p* = 0.02; 20 points), and VEGF and TNF-α (r = 0.67, *p* = 0.001; 20 points). The variation (i.e., Camp-Final *minus* Camp-Initial value) of VEGF correlated with IL-8 (r = 0.81, *p* = 0.048; 6 points) and TNF-α (r = 0.95, *p* = 0.004; 6 points).

**Figure 7:**
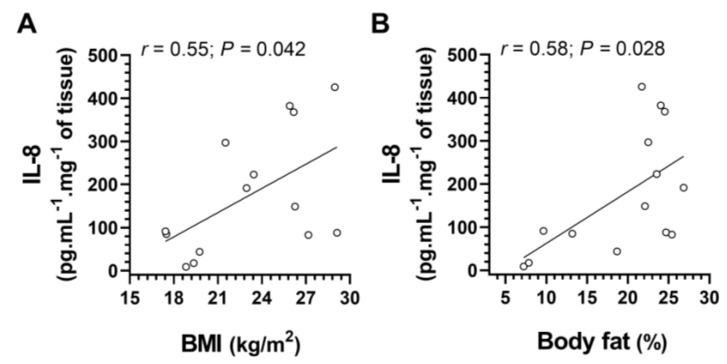
Correlations between (**A**) *Interleukin*-8 (IL-8) and body mass index (BMI), and (**B**) *Interleukin*-8 and body fat. The dots represent the individual datum. Number of samples = 14 for both correlations.

There was a correlation or a tendency to correlation for body mass with IL-8 and IL1-β (for IL-8: r = 0.52, *p* = 0.057; for IL-1b: r = 0.59, *p* = 0.025; 14 points for both); wherein IL-8 also presented a positive correlation with BMI (r = 0.55, *p* = 0.042; 14 points) (Figure 7A) and body fat (r = 0.58, *p* = 0.028; 14 points) (Figure 7B).

### 3.3. 7-Week Camp Changes TSH and T4

**Figure 8:**
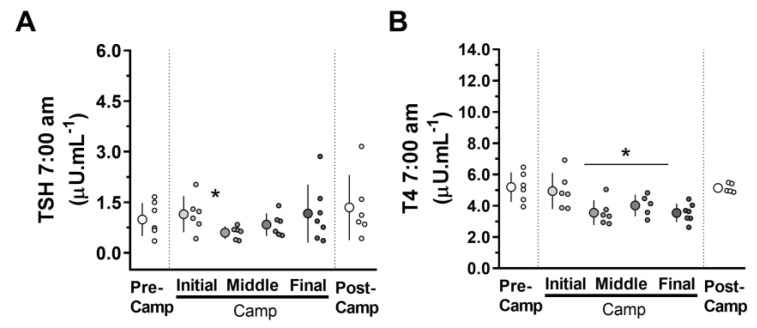
Hormonal concentrations measured at the Pre-Camp (i.e., 2nd and 3rd days on the ship, before camp; white circles), at Camp [Initial (i.e., 4th day in camp), in the Middle (19th and 33rd days in camp), and at Final (i.e., 45th day in camp) moments at camp; grey circles], and at the Post-Camp (i.e., 4th day on the ship, after camp; white circles) during an Antarctic expedition: (**A**) Thyroid-stimulating hormone (TSH), (**B**) Thyroxine (T4), at 7:00 am. The data are expressed as mean ± SD. The dots represent the individual data. * Significantly different (*p* < 0.05); for TSH, different from Post-Camp; for T4, different from the Pre-Camp moment. TSH, n = 6 since the data of an outlier individual were excluded. T4, n = 7.

For TSH, a difference was observed between the moments (*F* = 2.882; *p* = 0.034; *ES* = 0.8), with lower values during Camp-Middle (i.e., 19 days of camp) in relation to the Post-Camp (*p* = 0.03), with a tendency to difference for the Camp-Final (*p* = 0.08). There was also a large difference for T4 between the moments (*F* = 13.103; *p* = <0.001; *ES* = 2.0), with lower values during Camp-Middle (i.e., 20th day and 33rd day of camp) and Camp-Final in relation to the Pre-Camp and Post-Camp (*p* = ≤0.001, for all comparisons) (Figure 8).

### 3.4. 7-Week Camp Increase Urinary Catecholamines

**Figure 9:**
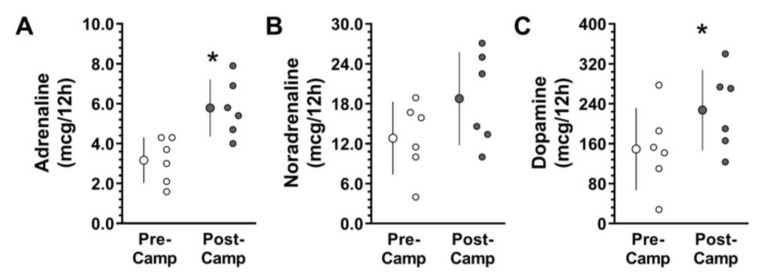
Twelve-hour (overnight) urinary catecholamine concentration was measured at the Pre-Camp (i.e., on the ship, before camp; white circles) and Post-Camp (i.e., on the ship, after camp; grey circles) moments: (**A**) Adrenaline, (**B**) Noradrenaline, (**C**) Dopamine. n = 6 due to an outlier exclusion. The data are expressed as mean ± SD. The dots represent the individual data. * Significantly different (*p* < 0.05) from the Pre-Camp.

There was an increase in total catecholamines in Post-Camp compared to Pre-Camp (*p* = 0.04; *ES* = 1.0) due to the increase in adrenaline (*p* = 0.009; *ES* = 2.0) (Figure 9A), dopamine (*p* = 0.05; *ES* = 0.9) (Figure 9C) and a tendency to increase in noradrenaline (*p* = 0.09; *ES* = 0.9) (Figure 9B).

### 3.5. Cardiac Autonomic Control

For the time-domain data, a reduction in Mean RR was observed in Post-Camp compared to Camp-Final and Camp-Middle moments, reflecting the difference observed for Mean HR (Table 3). For NNxx and pNNxx, there was a trend toward lower values with moderate effect in Post-Camp. For the frequency-domain, LF/HF ratio tended to increase in the Post-Camp. No changes were observed in other HRV parameters.

### 3.6. Characterization of the Dietary Pattern

The average daily total energy intake (TEI) was 2561 kcal, of which 23.8 ± 11.6% was natural or minimally processed foods, 29.4 ± 15.1% processed foods, and 45.2 ± 17.7% ultra-processed foods (for macronutrient analysis see Supplemental material S2, Table S2).

## 4. Discussion

A seven-week Brazilian camp in Antarctica altered the salivary, hormones, and markers of inflammation. We observed a decrease in the levels of *Pseudomonadota* (former *Proteobacteria)* and an increase in the pro-inflammatory markers IL-8 and leptin but reduced VEGF.

In the present study, salivary microbiome phyla predominance agrees with previous descriptions of major salivary microbiome phyla in three healthy male adults [51] and during an expedition including an Antarctic Sea voyage and a stay at the Indian Antarctic station [52].

Camping in Antarctica resulted in a reduction in *Pseudomonadota*. Common human pathogens found in the *Pseudomonadota* phylum are increased in metabolic disorders [51,52] and are described as often overrepresented during inflammatory conditions [53]. However, the reduction of *Pseudomonadota* occurred despite increases in pro-inflammatory markers IL-8 and leptin. Shin et al. [54] point out that *Pseudomonadota* is a front-line responder and susceptible to environmental factors. *Pseudomonadota* is exercise-responsive, with physical training decreasing its abundance in humans [55] and rats [56]. Permanence in the Antarctic field to carry out prospecting activities—similar to that carried out by the expeditioners of the present expedition—improved the physical performance of untrained individuals in a previous study [44]. Thus, it is possible that physical effort in the camp reduced the *Pseudomonadota* population. In fact, there was an increase in fat-free mass (as observed in Table 1), pointing to a muscle mass adaptation to effort load (i.e., hypertrophy). It is also necessary to consider that *Pseudomonadota* is present in the natural environment, including air and soil [54]. Since the oral cavity is the initial portion of the digestive tract and is in contact with the exterior, the Antarctic natural environment is a potential modulator of the salivary microbiome.

Given the low supply of fresh food under camping conditions in Antarctica, we expected a reduction in the abundance and diversity of the microbiome. In fact, %TEI of processed and ultra-processed foods was ~3-fold greater than in natura and minimally processed foods, an inverse outcome to that found in the Brazilian population, for which consumption of in natura and minimally processed foods is ~2-fold greater than processed and ultra-processed foods [57]. However, the 50 days of camping in Antarctica did not change the Shannon, Chao-1, and Simpson indices. We speculate that the contact with the new environment and the physical effort routine may have contributed to the maintenance of these indices. However, it is also necessary to consider that the individuals remained on a ship prior to field disembarkation. The ship already imposes confinement conditions, and where the availability and variety of fresh food are not usual, which may have influenced the microbiome even before the field period.

At the microbiome genus level, a biphasic pattern of relative abundance was observed with a significant difference for *Granulicatella* and *Bergeyella*, first increasing and then reducing at the end of the period in the field. This pattern indicates an initial environment-dependent change and later adaptation. The Gram-negatives *Neisseria*, *Pasteurellaceae-unclassified*, and the Gram-positive *Ruminococcaceae*-[G-1] showed or tended to show main changes at the end of the camp (i.e., Camp-Final). Tea consumption changes the microbiome [58], and in camp, it improves the thermal sensation. Given the routine of hot tea consumption in the field (morning, during field trips, and at the end of the day), it is a potential contributor to influencing the microbiome. As in the present study, Jin et al. [59] evaluated fecal samples and reported the influence of field stay in Antarctica on microbiome changes. In contrast to our study, Bhushan et al. [52] investigated the influence of 25 days of Antarctic Sea voyage and 30 days of stay at an Antarctic station on oral microbiome and reported increased diversity and an enhanced abundance of *Pseudomonas* spp. after the sea voyage and also at the end of 30 days at the station. The difference between our findings and those obtained by Bhushan et al. [52] points to the impact of the different conditions of permanence (ship, research station, or camps) in Antarctica on the microbiome.

The permanence in the Antarctic field tended to augment adipocyte-derived leptin and neutrophil chemotactic factor IL-8 without increases in the other pro-inflammatory cytokines [60,61,62]. Weight gain and diet patterns affect these markers, and body fat is an predictor of both [63,64]. In the present study, IL-8 showed a positive correlation with BMI and body fat, corroborating the relationship between body mass gain and inflammation; regardless, body mass and fat percentage did not change during camp. Schueller et al. [65] investigated gingival epithelial cells and suggested that a higher inflammatory activity may be linked to stimulation by bacterial endotoxins. In this sense, leptin presented a tendency to have positive correlations with *Bacilotta*. Although IL-8 did not show a specific correlation with any bacterial phyla, leptin promotes the expression of IL-8 [62]. Furthermore, it is plausible to consider that leptin and IL-8 reflected inflammation derived from meals [66,67]. Specifically, the intake of ultra-processed foods in camp, rich in simple carbohydrates and saturated fats, may have stimulated the production of reactive oxygen species and consequent increase of leptin and IL-8 levels [58], modulating the inflammatory response. Also, leptin and IL-8 contributes to body weight control due to anorexigenic effects at the central nervous system [62,68].

Stress is an additional factor to trigger IL-8 increases [69]. Although we observed a higher concentration of catecholamines at the end of the expedition, this stress-related measurement was performed only in the moments on the ship (Pre- and Post-Camp). During the period in the field, we accessed HRV as an indicator cardiac autonomic regulation. We did not observe changes in the LF/HF ratio (an indicator of the predominance of cardiac sympathetic activity) during the camp. Furthermore, no changes were observed in salivary IL-6, IL-10, and TNF-α, which also had a significant stress-related effect [69,70]. Based on the above-mentioned points, we tend to discard the physiological stress to explain the increase in IL-8. However, the IL-8 peak effect occurs, on average, after 85 min (and in a maximum of 240 min) of the stressor stimulus, presenting for the measured cytokines the longest time between the acute stimulus of stress and the peak of response [69]. One possibility is that the stress of the night in Antarctica, with winds that ‘beat’ the tent and light exposure during part of the sleep period, has stimulated the IL-8. Thus, IL-8 may be a morning marker of acute nocturnal stress response experienced due to Antarctic environmental conditions. Future studies that evaluate HRV and stress-related hormone markers during the night period will contribute to the elucidation of the issue.

Despite the tendency to increase IL-8 and no change in IL-1 and IL-6, we observed a reduction of VEGF. Takeyama et al. [71] showed in an in vitro assay the decrease of VEGF in retinal epithelium with decreased temperature. In the same sense, Gagnon et al. [72] reported lower VEGF during 60 min of exercise in cold without shivering (0 °C) compared to thermoneutral conditions (22 °C), suggesting that the low temperatures in the Antarctica may have suppressed VEGF. Takeyama et al. [71] suggested that a decrease in VEGF expression correlates with decreased metabolism in human retinal epithelial cells cultured under hypothermia. However, other studies report that cooling upregulates mRNA expression of angiogenic factors in adipose and skeletal muscle tissues, including VEGF [73,74]; also, shivering increases VEGF during exercise in cold, compared with non-shivering in cold conditions [72].

Nevertheless, to the best of our knowledge, no study investigated the mechanisms underlying cold effects, specifically on human salivary VEGF. Thus, exposure to the cold effect on salivary VEGF is an issue to be investigated. Additionally, the inverse correlation between VEGF and *Actinomycetota* (former *Actinobacteria*) reveals another candidate to affect VEGF. VEGF is implicated in the healing processes and protects the mucosa from physical, chemical, and bacterial potential deleterious factors [75,76]. Considering the possible impaired regeneration and disrupted epithelial integrity, future studies should investigate the role of VEGF suppression on the risk of development of oral pathologies in similar camp conditions and other ICE.

Antarctic summer camp induced a biphasic TSH response, with an initial reduction followed by an increase during the field period. The TSH reduction reinforced the influence of natural luminosity and albedo of the snow on this hormone [77,78] and was also reported in a previous summer camp in Antarctica [1]. A cold-induced increase in TSH can explain the subsequent return to initial levels [26,79]. T4 reduction possibly reflects increases in T4 tissue uptake and T4-to-T3 conversion [26,80] via the iodothyronine deiodinase D2 (thyroxine-5’-deiodinase II) [81,82] since cold exposure induces D2 activity. T4 tissue uptake and T4-to-T3 conversion enhance heat production through mitochondria calorigenic effect [81] and are reported as an adaptation to cold acclimatization [26,79]. The simultaneous increase in TSH and a decline in T4 was shown by Reed et al. [83] over a year in Antarctica, which also reported a raised resting metabolic rate. It is also noteworthy the role of leptin in energy expenditure [84]. During the Antarctic camp stay thyroid hormone patterns and augmented leptin suggest an increased basal metabolic rate- this hypothesis should also be tested, as we have not measured T3 or oxygen consumption in the present study.

LF/HF ratio elevation in the Post-Camp moment on the ship, simultaneously with the catecholamines, reveals a higher activity of the sympathetic axis. These responses may reflect the demands for leaving the camp and returning to the ship. On the other hand, it is necessary to consider that the measurements were carried out after crossing the Drake Passage—when turbulent waters increase the ship’s balance; thus, increased sympathetic activity may result from acute stress.

We accessed changes in the microbiome, inflammatory markers, and metabolic hormones profiles in the context of “real life” [85]—in one of the extremes on the planet—under natural environment and social context, with repeated intra-individual measurements. However, the current study presents defies and limitations of studies carried out in the field, including some unexplored variables that can influence oral environment. Our volunteers presented a certificate issued by a dentist to start the expedition, but we did not control the volunteers’ procedures during routine oral cavity hygiene. Considering the importance of oral hygiene maintenance and plaque removal [86,87] and as mouthwashes affect the amount of microbial plaque [88], the individual hygiene procedures of the oral cavity could be an intervening factor in the present study. Nevertheless, the similar field routine between individuals (including toothbrushing times in the morning, before leaving for the field, and at night, after the last meal) may have attenuated individual differences in oral cavity hygiene routine. Additionally, the use of probiotics [89] and natural compounds [90] can modify clinical aspects and microbiological milieu in periodontal patients; thus, once the use of these substances was not controlled, these compounds could have an effect in the volunteers studied in the present report. In future studies, we suggest (i) standardizing oral cavity hygiene procedures, (ii) explore the use of probiotics and natural compounds and (iii) determining whether changes in the microbiome pattern are transient or generate longer-lasting impacts on health.

Furthermore, as field expeditions in Antarctica are usually composed of few people, the non-homogeneous reduced sample is a limiting factor of the present study. However, because of the logistics of expeditions, we consider that evaluating a group composed of 7 individuals represents a moderate size group since the number of subjects in camps under extreme conditions usually varies from 4 until about 10 to 14 individuals [1,3,51,91,92,93] Also, reporting physiological field modifications does not reveal any cause-effect relationship but shows patterns of changes that can provide the basis for future investigations under controlled laboratory conditions. Moving from this perspective, we emphasize the exploratory aspects of the present study to construct posterior hypotheses.

## 5. Conclusions

Staying in a long-term camp in Antarctica resulted in fluctuations in the microbiome, with transient shifts of *Granulicatella*, *Ruminococcaceae*-[G-1], and *Bergeyella* and reduction of *Pasteurellaceae-unclassified*. At the phylum level, the changes reduced *Pseudomonadota*. Concurrently, pro-inflammatory markers IL-8 and leptin tended to increase, while the angiogenic factor VEGF decreased, suggesting that distinct environmental factors (natural and habits) modulated general and oral inflammation. The end of the expedition represented the moment of higher physiological stress, possibly due to the demands for dismantling and removal from the camp or the acute effect experienced by the ship’s balance.

## Figures and Tables

**Table 1:** Anthropometric characteristics of the volunteers in Initial and Final moments in camp.

	Camp			
*n* = 7	Initial	Final	*p*-Value	*ES*
Body mass (kg)	69.8 ± 16.2	70.3 ± 14.9	0.49	0.03
∑Skinfold (mm)	128.0 ± 49.2	117 ± 39.7	0.19	0.25
Body fat (%)	20.1 ± 7.4	18.8 ± 6.9	0.26	0.18
Free-fat body mass (kg)	55.2 ± 10.6	56.6 ± 10.3 *	0.01	0.13
Fat body mass (kg)	14.6 ± 7.4	13.6 ± 6.4	0.29	0.14

* Significantly different (*p* < 0.05) from the Initial moment. The data are expressed as mean ± SD.

**Table 2:** Salivary Interleukin-6 at 7:00 am and 7:00 pm at the Pre-Camp (i.e., 2nd and 3rd days on the ship, before camp), and Camp [Initial (i.e., 4th day in camp), and Final (i.e., 45th day in camp) moments at camp] during an Antarctic expedition.

	Pre-Camp	Camp		Post-Camp	One-Way ANOVA RM	
		Initial	Final			*ES*
IL-6 7:00 am (pg.mL^−1^.mg^−1^)	34.11 ± 25.00	36.10 ± 35.99	27.76 ± 11.84	37.85 ± 25.42	*F* = 0.15 *p* = 0.93	0.3
IL-6 7:00 pm (pg.mL^−1^.mg^−1^)	30.32 ± 19.06	44.45 ± 17.65	41.58 ± 17.09	46.46 ± 51.43	*F* = 0.41 *p* = 0.75	0.4

The data are expressed as mean ± SD. An outlier individual was excluded from IL-6 analysis.

**Table 3:** Heart rate variability (HRV) parameters measured at the Pre-Camp (i.e., 2nd and 3rd days on the ship, before camp), at Camp [Initial (i.e., 5th day in camp), in the Middle (20th and 34th days in camp), and at Final (i.e., 46th day in camp) moments at camp], and the Post-Camp (i.e., 4th day on the ship, after camp) during an Antarctic expedition. The data are expressed as mean ± SD. ^M^ Moderate effect size. ^L^ Large effect size. * Significantly different (*p* < 0.05); for Mean RR and Mean HR, different from Camp-Final and Camp-Middle (34th day in camp).

		Camp						
	Pre-Camp	Initial	Middle 20th Day	Middle 34th Day	Final	Post-Camp	One-Way ANOVA RM	
								*ES*
Time-domain								
Mean RR (ms)	877.7 ± 204.2	846.8 ± 199.4	853.3 ± 153.7	911.0 ± 143.6	915.9 ± 154.1	839.8 * ± 136.2	*F* = 2.784 *p* = 0.036	0.5
SDNN (ms)	45.2 ± 17.9	36.1 ± 13.0	35.5 ± 8.8	40.8 ± 16.8	43.3 ± 10.0	37.8 ± 16.0	*F* = 1.030 *p* = 0.419	0.6 ^M^
Mean HR (beats/min)	71.1 ± 14.1	73.9 ± 14.9	72.0 ± 12.2	67.1 ± 10.3	67.3 ± 11.4	73.2 * ± 11.9	*F* = 3.051 *p* = 0.025	0.6 ^M^
STD HR (beats/min)	35.7 ± 11.7	30.7 ± 7.6	29.7 ± 4.2	30.5 ± 13.0	32.2 ± 7.6	35.8 ± 17.5	*F* = 1.088 *p* = 0.388	0.5
Min HR (beats/min)	63.2 ± 11.5	67.7 ± 14.3	65.9 ± 10.3	61.4 ± 9.8	60.8 ± 9.8	64.9 ± 9.9	*F* = 2.213 *p* = 0.080	0.5
Max HR (beats/min)	81.6 ± 16.3	84.7 ± 17.0	82.1 ± 15.0	77.2 ± 14.4	77.3 ± 12.8	84.4 ± 15.3	*F* = 2.515 *p* = 0.052	0.5
RMSSD (ms)	47.4 ± 27.7	34.8 ± 20.1	35.6 ± 16.7	43.1 ± 18.6	48.0 ± 15.8	38.6 ± 27.5	*F* = 0.873 *p* = 0.510	0.6 ^M^
Ln (RMSSD)	1.61 ± 0.24	1.49 ± 0.23	1.52 ± 0.18	1.60 ± 0.18	1.66 ± 0.15	1.50 ± 0.29	*F* = 1.411 *p* = 0.250	<0.1
NNxx	70.3 ± 59.5	48.7 ± 50.9	50.71 ± 52.0	72.7 ± 52.3	85.6 ± 53.9	32.8 ± 35.2	*F* = 2.173 *p* = 0.085	0.8 ^M^
pNNxx (%)	23.2 ± 23.4	16.3 ± 20.9	16.6 ± 19.1	23.2 ± 17.6	27.9 ± 20.0	9.3 ± 9.7	*F* = 2.144 *p* = 0.089	1.1 ^M^
Frequency-domain								
LF (ms^2^)	770.8 ± 340.7	763.9 ± 458.4	536.3 ± 253.9	755.5 ± 859.7	728.1 ± 331.3	740.7 ± 527.6	*F* = 0.312 *p* = 0.902	0.4
HF (ms^2^)	1028.9 ± 1132.1	654.8 ± 622.6	622.7 ± 481.8	964.0 ± 1010.8	957.0 ± 552.9	734.6 ± 990.4	*F* = 0.470 *p* = 0.796	0.4
LF/HF	1.49 ± 1.10	1.48 ± 0.82	1.21 ± 0.90	0.90 ± 0.49	0.94 ± 0.63	2.87 ± 2.57	*F* = 2.301 *p* = 0.071	1.1 ^M^

## Data Availability

The data presented in this study are available in Supplementary materials S2 and S4 and are available on request from the corresponding author.

## Supplementary Materials

The following supporting information can be downloaded at: https://www.mdpi.com/article/10.3390/microorganisms11020339/s1. Supplemental Materials S1: Model of medical certificate to enable participation in the Antarctic expedition; S2: Consumption of natural or minimally processed foods, processed foods, and ultra-processed foods obtained by 24 h food recall questionnaire (FRQ) in the camp in the Antarctic; S3: Values obtained in normality and equivariance tests of statistical analysis for posterior pairwise comparisons; S4: Individual relative abundance of microbiome phyla and species detected during a camp in Antarctica; S5: Association between the relative abundance of bacterial genus and diversity indexes with cytokine and signal proteins. References are cited in [94,95]
