# Supplementary material for: A 7-Week Summer Camp in Antarctica Induces Fluctuations on Human Oral Microbiome, Pro-Inflammatory Markers and Metabolic Hormones Profile
Source: Microorganisms. 2023 Jan 30;11(2):339. doi: 10.3390/microorganisms11020339 (PMC9960157; doi:10.3390/microorganisms11020339)
Supplement: Supplementary file 1 [file microorganisms-11-00339-s001.zip › _.Suppl Material 1.pdf]

Model of medical certificate to enable participation in the Antarctic expedition

Michele M Moraes, Thiago T Mendes, Leandro Borges, Alice L Marques, Cristian Núñez-Espinosa, Dawit AP Gonçalves, Carolina B Simões, Tales S Vieira, Roberto VP Ladeira, Talita GB Lourenco, Danielle V Ribeiro, Elaine Hatanaka, Debora Heller, Rosa ME Arantes

This is Supplemental material S1 for the article: **A 7-week summer camp in Antarctica induces fluctuations on human oral microbiome, pro-inflammatory markers and metabolic hormones profile**

To participate in the expedition, the individuals needed to present a medical certificate indicating that they "were physically and mentally healthy to exercise professional activities in a location deficient in health care and with occasional exposure to adverse weather conditions (intense cold)", and not presenting any of the disabilities listed below.

- Systemic arterial hypertension in any form (indices greater than 140 x 90 mmHg), uncontrolled.
- Arteries, veins or lymphatic affections that cause circulation deficiencies.
- All acute or chronic affections, compensated or not, of the heart and vessels.
- Acute and chronic affections of the lips, tongue, oral mucosa, salivary glands, pharynx and lesions that cause disturbances in mastication and swallowing.
- Acute or chronic affections, even asymptomatic and subclinical, of the entire digestive tract, including the liver, biliary tract and pancreas.
- Any disease that requires a diet, incompatible with Antarctica.
- Any disease or injury to the respiratory system that reduces respiratory capacity.
- Any form of pharyngitis, asthma, bronchitis or tuberculosis, including sequelae.
- Acute and chronic conditions that compromise the locomotor system and the spine.
- Fractures that are not consolidated or healed in a vicious position, which interfere with the normal function of the limb.
- Functional absence of a kidney.
- Any neurological pathology, including epilepsy in all its forms, neuromyopathies and cerebral parasitic diseases.
- Acute or chronic recurrent sinusopathies.
- Any pathology of the outer, middle and inner ear.
- Glaucoma (even if intraocular pressure is stable, under treatment) or intraocular pressure above 16 mmHg for any age.
- Any endocrinopathy.
- Any psychiatric pathology, with or without regular, current or past treatment.
- Traumatic brain injury for 2 years or less;
- Drug abuse without dependence.
- Drug addiction, including alcoholism.
