# Supplementary material for: A 7-Week Summer Camp in Antarctica Induces Fluctuations on Human Oral Microbiome, Pro-Inflammatory Markers and Metabolic Hormones Profile
Source: Microorganisms. 2023 Jan 30;11(2):339. doi: 10.3390/microorganisms11020339 (PMC9960157; doi:10.3390/microorganisms11020339)
Supplement: Supplementary file 1 [file microorganisms-11-00339-s001.zip › _.Suppl Material 2.pdf]

Consumption of natural or minimally processed foods, processed foods and ultra-processed foods obtained by 24h-food recall questionnaire (FRQ) in the camp in Antarctica

Michele M Moraes, Thiago T Mendes, Leandro Borges, Alice L Marques, Cristian Núñez-Espinosa, Dawit AP Gonçalves, Carolina B Simões, Tales S Vieira, Roberto VP Ladeira, Talita GB Lourenco, Danielle V Ribeiro, Elaine Hatanaka, Debora Heller, Rosa ME Arantes

This is Supplemental material S2 for the article: **A 7-week summer camp in Antarctica induces fluctuations on human oral microbiome, pro-inflammatory markers and metabolic hormones profile**

Table S2. Absolute and relative consumption of natural or minimally processed foods, processed foods and ultra-processed foods in the camp in Antarctica

	Total food	Natural or minimally processed foods	Processed foods	Ultra-processed foods
**Kcal/day**	2561.7 ± 1183.8	549.7 ± 281.6	759.4 ± 597.6	1213.9 ± 826.1
**% of total energy intake (TEI)**	100	23.8 ± 11.6	29.4 ± 15.1	45.2 ± 17.7
**CHO (g)**	320.5 ± 151.7	77.2 ± 52.2	60.6 ± 58.7	173.2 ± 122.0
**Fat (g)**	107.3 ± 54.6	18.4 ± 17.7	43.5 ± 34.9	45.3 ± 35.3
**PTN (g)**	84.5 ± 49.9	20.7 ± 12.7	34.9 ± 44.2	28.9 ± 15.6
**Fiber (g)**	20.3 ± 13.2	9.6 ± 11.6	3.8 ± 4.4	6.9 ± 2.8

Footnote: The application of the 24h-food recall questionnaire (FRQ) to characterize the dietary pattern was carried out along the Middle and Final period. Because there was no differentiation between weekdays and weekends in the camp routine, the registration days were random. The macronutrient analysis was carried out using the Brazilian Food Composition Table (TACO) [94] that was included in the software. For foods not included in the database of the software, TACO database was updated using the packaged food labels. The data are expressed as mean ± SD. CHO: carbohydrate. PTN: protein. Data obtained from 20 FRQ of the seven volunteers (minimum one record and maximum four records *per* volunteer).

Reference

94. Lima, D.M.; Padovani, R.M.; Rodriguez-Amaya, D.B.; Farfán, J.Á.; Nonato, C.T.; Lima, M.T.; Salay, E.; Colugnati, F.A.B.; Galeazzi, M.A.M. *Tabela Brasileira de Composição de Alimentos* (*TACO*), 4th ed.; NEPA-Unicamp: Campinas, Brazil, 2011. Available online: https://www.cfn.org.br/wp-content/uploads/2017/03/taco_4_edicao_ampliada_e_revisada.pdf (accessed on 3 July 2022).
