# Supplementary material for: A 7-Week Summer Camp in Antarctica Induces Fluctuations on Human Oral Microbiome, Pro-Inflammatory Markers and Metabolic Hormones Profile
Source: Microorganisms. 2023 Jan 30;11(2):339. doi: 10.3390/microorganisms11020339 (PMC9960157; doi:10.3390/microorganisms11020339)
Supplement: Supplementary file 1 [file microorganisms-11-00339-s001.zip › _.Suppl Material 3.pdf]

Values obtained in normality and equivariance tests of statistical analysis for posterior pairwise comparisons

Michele M Moraes, Thiago T Mendes, Leandro Borges, Alice L Marques, Cristian Núñez-Espinosa, Dawit AP Gonçalves, Carolina B Simões, Tales S Vieira, Roberto VP Ladeira, Talita GB Lourenco, Danielle V Ribeiro, Elaine Hatanaka, Debora Heller, Rosa ME Arantes

This is Supplemental material S3 for the article: **A 7-week summer camp in Antarctica induces fluctuations on human oral microbiome, pro-inflammatory markers and metabolic hormones profile**

Table S3.1. Values obtained in normality and equivariance tests at the phylum level

Phylum	Normality	Equal Variance
Actinomycetota	W-Statistic = 0.934 P = 0.181	P = 0.604
Bacteria_unclassified*	W-Statistic = 0.956 P = 0.473	P = 0.618
Bacteroidota	W-Statistic = 0.963 P = 0.606	P = 0.747
Bacillota Firmicutes	W-Statistic = 0.935 P = 0.196	P = 0.284
Fusobacteria	W-Statistic = 0.953 P = 0.421	P = 0.868
Gracilibacteria_(GN02) T: reciprocal	W-Statistic = -- P = 1.000	P = 0.867
Pseudomonadota Proteobacteria	W-Statistic = 0.982 P = 0.962	P = 0.607
SR1 T: sqrt	W-Statistic = 0.975 P = 0.849	P = 0.466
Spirochaetes T: log10	W-Statistic = 0.958 P = 0.513	P = 0.589
Synergistetes T: reciprocal	W-Statistic = -- P = 1.000	P = 0.626

Footnote: *Pseudomonadota*, former *Proteobacteria*. *Bacillota*, former *Firmicutes*. *Bacteroidota,* former *Bacteroidetes*. *Actinomycetota*, former *Actinobacteria*. T: transformation applied to the data. Sqrt: Square root.

Table S3.2. Values obtained in normality and equivariance tests at the genus level

Genus	Normality	Equal Variance
Clostridiales_unclassified T: ln	W-Statistic = 0.974 P = 0.835	P = 0.606
Granulicatella T: ln	W-Statistic = 0.939 P = 0.228	P = 0.295
Mogibacterium T: rank	W-Statistic = 0.947 P = 0.320	P = 0.198
Firmicutes_unclassified	W-Statistic = 0.979 P = 0.927	P = 0.630
Ruminococcaceae_[G-1] 1.0/	W-Statistic = 0.821 P = 0.002	P = 0.601
Veillonellaceae_[G-1]	Kruskal-Wallis	
Abiotrophia	W-Statistic = 0.946 P = 0.307	P = 0.501
Lachnoanaerobaculum	W-Statistic = 0.919 P = 0.096	P = 0.221
Solobacterium T: rank	W-Statistic = 0.954 P = 0.423	P = 0.880
Gemella T: sqrt	W-Statistic = 0.904 P = 0.050	P = 0.348
Filifactor	Kruskal-Wallis	
Oribacterium T: ln	W-Statistic = 0.967 P = 0.688	P = 0.340
Streptococcus T: ln	W-Statistic = 0.967 P = 0.684	P = 0.317
Ruminococcaceae_[G-2] T: rank	W-Statistic = 0.951 P = 0.381	P = 0.601
Lachnospiraceae_[XIV]_unclassi T: rank	W-Statistic = 0.956 P = 0.473	P = 0.790
Peptostreptococcaceae_[XI][G-5	Kruskal-Wallis	
Lactobacillus T: 1.0/	Kruskal-Wallis	
Veillonella	W-Statistic = 0.945 P = 0.293	P = 0.263
Dialister T: rank	W-Statistic = 0.960 P = 0.539	P = 0.368
Catonella T: rank	W-Statistic = 0.960 P = 0.551	P = 0.701
SR1_[G-1] T: rank	W-Statistic = 0.959 P = 0.516	P = 0.099
Sneathia	Kruskal-Wallis	
Leptotrichia	W-Statistic = 0.964 P = 0.635	P = 0.547
Fusobacterium T: ln	W-Statistic = 0.960 P = 0.544	P = 0.991
Bergeyella T: ln	W-Statistic = 0.965 P = 0.645	P = 0.444
Alloprevotella	W-Statistic = 0.969 P = 0.741	P = 0.268
Bacteroidetes_unclassified	W-Statistic = 0.962 P = 0.583	P = 0.810
Capnocytophaga	W-Statistic = 0.907 P = 0.057	P = 0.534
Prevotella	W-Statistic = 0.933 P = 0.178	P = 0.988

Bacteroides	W-Statistic = 0.980 P = 0.932	P = 0.137
Bacteroidales_unclassified T: ln	W-Statistic = 0.965 P = 0.649	P = 0.456
Actinomyces T: ln	W-Statistic = 0.953 P = 0.411	P = 0.410
Corynebacterium T: ln	W-Statistic = 0.979 P = 0.916	P = 0.923
Actinobacteria_unclassified T: ln	W-Statistic = 0.919 P = 0.096	P = 0.326
Rothia T: ln	W-Statistic = 0.949 P = 0.346	P = 0.749
Kingella T: ln	W-Statistic = 0.948 P = 0.335	P = 0.503
Eikenella T: rank	W-Statistic = 0.946 P = 0.309	P = 0.603
Neisseriaceae_unclassified T: rank	W-Statistic = 0.960 P = 0.539	P = 0.341
Neisseria	W-Statistic = 0.946 P = 0.312	P = 0.825
Escherichia T: rank	W-Statistic = 0.953 P = 0.412	P = 0.909
Stenotrophomonas T: ln	Kruskal-Wallis	
Treponema	W-Statistic = 0.958 P = 0.513	P = 0.589
Fretibacterium	Kruskal-Wallis	
Porphyromonas T: ln	W-Statistic = 0.981 P = 0.943	P = 0.715
Bacteroidales_[G-2] T: asinsqrt	W-Statistic = 0.936 P = 0.198	P = 0.943
Bacteroidetes_[G-5]	Kruskal-Wallis	
Tannerella T: ln	W-Statistic = 0.938 P = 0.224	P = 0.507
Megasphaera T: ln	W-Statistic = 0.963 P = 0.605	P = 0.093
Peptostreptococcaceae_[XI][G-7 T: rank	W-Statistic = 0.935 P = 0.189	P = 0.529
Stomatobaculum	W-Statistic = 0.944 P = 0.290	P = 0.816
Selenomonas ln	W-Statistic = 0.921 P = 0.104	P = 0.424
Peptostreptococcus	W-Statistic = 0.952 P = 0.400	P = 0.495
Lachnospiraceae_[G-3] T: rank	W-Statistic = 0.920 P = 0.099	P = 0.708
Peptostreptococcaceae_[XI][G-1 T: rank	W-Statistic = 0.955 P = 0.449	P = 0.127
Parvimonas rank	W-Statistic = 0.960 P = 0.541	P = 0.487
Staphylococcus T: ln	W-Statistic = 0.952 P = 0.392	P = 0.675
Atopobium T: rank	W-Statistic = 0.960 P = 0.543	P = 0.693
Actinobaculum	Kruskal-Wallis	
Campylobacter	W-Statistic = 0.929 P = 0.146	P = 0.537
Cardiobacterium T: ln	W-Statistic = 0.934 P = 0.187	P = 0.355
Haemophilus	W-Statistic = 0.929 P = 0.147	P = 0.951
Aggregatibacter T: rank	W-Statistic = 0.960 P = 0.551	P = 0.114
Pasteurellaceae_unclassified T: ln	W-Statistic = 0.981 P = 0.947	P = 0.631
Lautropia T: ln	W-Statistic = 0.982 P = 0.953	P = 0.498

Footnote: T: transformation applied to the data. Asinsqrt: Arcsin square root. Ln: Logarithmic. Rank: data replacement by their ranks (or average ranks in case of ties) - i.e., for the lowest value: rank 1; for the second lowest value: rank 2, and so on [95]. 1.0/: Reciprocal. Sqrt: Square root.

Reference

95. Conover, W. The rank transformation—an easy and intuitive way to connect many nonparametric methods to their parametric counterparts for seamless teaching introductory statistics courses. *WIREs Comput. Stat.* **2012**, *4*, 432–438. https://doi.org/10.1002/wics.1216.
