# Supplementary material for: A 7-Week Summer Camp in Antarctica Induces Fluctuations on Human Oral Microbiome, Pro-Inflammatory Markers and Metabolic Hormones Profile
Source: Microorganisms. 2023 Jan 30;11(2):339. doi: 10.3390/microorganisms11020339 (PMC9960157; doi:10.3390/microorganisms11020339)
Supplement: Supplementary file 1 [file microorganisms-11-00339-s001.zip › _.Suppl Material 4.pdf]

Individual relative abundance of microbiome phyla and species detected during a camp in Antarctica

Michele M Moraes, Thiago T Mendes, Leandro Borges, Alice L Marques, Cristian Núñez-Espinosa, Dawit AP Gonçalves, Carolina B Simões, Tales S Vieira, Roberto VP Ladeira, Talita GB Lourenco, Danielle V Ribeiro, Elaine Hatanaka, Debora Heller, Rosa ME Arantes

This is Supplemental material S4 for the article: **A 7-week summer camp in Antarctica induces fluctuations on human oral microbiome, pro-inflammatory markers and metabolic hormones profile**

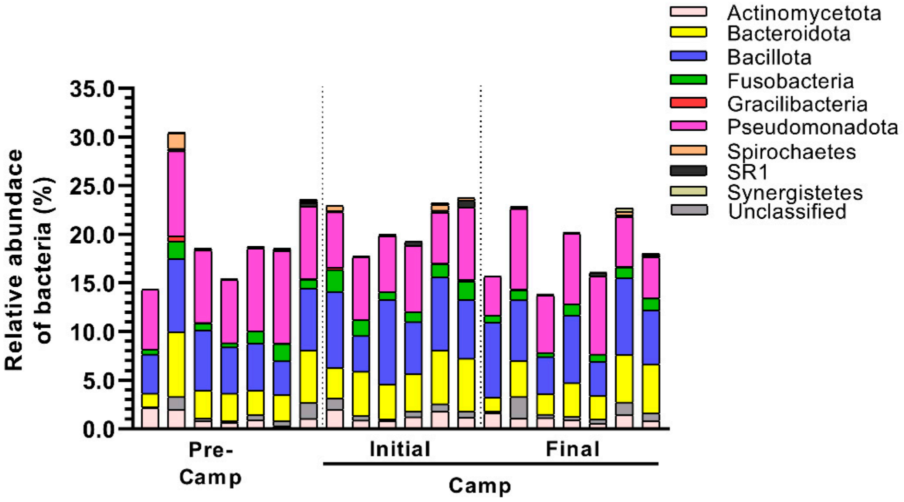

Figure S4. Microbiome phyla. *Pseudomonadota*, former *Proteobacteria*. *Bacillota*, former *Firmicutes*. *Bacteroidota,* former *Bacteroidetes*. *Actinomycetota*, former *Actinobacteria*. The data are expressed as individual values.

Table S4. Microbiome species detected.

Taxon	Pre-Camp	Camp	
		Initial	Final
Haemophilus_unclassified	0,0295036	0,017403	0,025236
Streptococcus sp,_oral_taxon_058	0,0111506	0,013206	0,011071
Streptococcus_unclassified	0,0080583	0,009853	0,009032
Haemophilus paraphrohaemolyticus	0,0087987	0,009641	0,007774
Neisseria subflava	0,0089107	0,007273	0,003639
Neisseria_unclassified	0,0063539	0,007397	0,004188
Prevotella_unclassified	0,0040473	0,005838	0,005328
Lautropia mirabilis	0,0048636	0,001411	0,003694
Veillonella_unclassified	0,0027089	0,003271	0,003005
Pasteurellaceae_unclassified	0,0043243	0,002233	0,002048
Pasteurellaceae_unclassified	0,0043243	0,002233	0,002048
Veillonella dispar	0,0020311	0,002589	0,003229
Gemella morbillorum	0,0018677	0,003336	0,002115
Porphyromonas pasteri	0,0024811	0,002768	0,00164
Veillonella parvula	0,0022533	0,002432	0,001971
Aggregatibacter_unclassified	0,0020957	0,001635	0,002475
Fusobacterium_unclassified	0,00153	0,003369	0,001551
Granulicatella adiacens	0,0015601	0,002122	0,002246
Fusobacterium periodonticum	0,0020027	0,002162	0,001023
Neisseria flava	0,001737	0,002168	0,00128
Prevotella oris	0,001809	0,001674	0,001422
Prevotella nanceiensis	0,0012907	0,001406	0,002063
Leptotrichia_unclassified	0,0017596	0,002177	0,000934
Kingella_unclassified	0,0010174	0,001522	0,002111
Granulicatella elegans	0,0014067	0,002531	0,000765
Actinomyces_unclassified	0,0013174	0,002212	0,001112
Oribacterium_unclassified	0,0011313	0,000622	0,002593
Neisseriaceae_unclassified	0,0010251	0,001732	0,001661
Neisseriaceae_unclassified	0,0010251	0,001732	0,001661
Lachnospiraceae_[XIV]_unclassified	0,001754	0,0013	0,00104
Streptococcus vestibularis	0,0005561	0,001057	0,001898
Bergeyella sp,_oral_taxon_900	0,0007763	0,002846	5,14E-05
Alloprevotella_unclassified	0,0009359	0,00101	0,001388
Rothia dentocariosa	0,001511	0,000587	0,001126

Prevotella sp,_oral_taxon_472	0,0011464	0,001464	0,000697
Rothia mucilaginosa	0,000879	0,001048	0,001315
Actinomyces sp,_oral_taxon_180	0,0011067	0,001761	0,00045
Bergeyella sp,_oral_taxon_322	0,0011979	0,000622	0,001188
Corynebacterium durum	0,0014711	0,001083	0,000484
Capnocytophaga_unclassified	0,0010576	0,001167	0,000826
Porphyromonas_unclassified	0,0010479	0,001081	0,000898
Capnocytophaga sputigena	0,0008656	0,0006	0,001422
Alloprevotella sp,_oral_taxon_473	0,0006491	0,001533	0,000786
Rothia aeria	0,001103	0,000835	0,000898
Megasphaera micronuciformis	0,0010571	0,000629	0,001042
Prevotella pallens	0,0003454	0,000676	0,001642
Alloprevotella tannerae	0,0005354	0,00163	0,000615
Atopobium_unclassified	0,0005033	0,00076	0,001333
Selenomonas_unclassified	0,0007573	0,000505	0,001255
Leptotrichia sp,_oral_taxon_392	0,000598	0,00097	0,000957
Porphyromonas sp,_oral_taxon_930	0,0008523	0,00118	0,000418
Campylobacter_unclassified	0,0009814	0,000487	0,000862
Streptococcus parasanguinis_II	0,0005316	0,000536	0,001263
Capnocytophaga leadbetteri	0,000583	0,001464	0,000403
aphrophilus	0,0011903	0,000598	0,000522
Prevotella salivae	0,000503	0,001358	0,000505
Veillonella sp,_oral_taxon_780	0,0008371	0,000682	0,000738
Catonella morbi	0,0008807	0,000758	0,000604
Porphyromonas catoniae	0,0008581	0,000547	0,000731
Treponema_unclassified	0,0010214	0,000817	0,000327
SR1_[G-1] sp,_oral_taxon_875	0,0005467	0,001163	0,000442
Leptotrichia hongkongensis	0,0005163	0,000582	0,000955
Peptostreptococcaceae_[XI][G-7]	0,0009093	0,000735	0,000406
Lachnoanaerobaculum umeaense	0,000334	0,000684	0,001012
Bacilli defectiva	0,0005847	0,000921	0,000554
Alloprevotella sp,_oral_taxon_308	0,0004346	0,000731	0,000835
Actinomyces sp,_oral_taxon_169	0,000596	0,000576	0,000801
Prevotella melaninogenica	0,000446	0,000742	0,00075
Corynebacterium matruchotii	0,0004554	0,000702	0,000752
Campylobacter rectus	0,0006607	0,000826	0,000401
Actinomyces graevenitzii	0,0003076	0,001322	0,000296

Ruminococcaceae_[G-1]	0,0005427	0,001145	0,000192
Ruminococcaceae_[G-1] sp,_oral_taxon_075	0,0005427	0,001145	0,000192
Prevotella veroralis	0,0009873	0,000357	0,000385
Prevotella denticola	0,0005181	0,000538	0,000689
Kingella oralis	0,0004023	0,000848	0,000535
Eikenella corrodens	0,0007556	0,000647	0,000317
Dialister invisus	0,0006436	0,000715	0,000362
Cardiobacterium hominis	0,0006377	0,000485	0,000543
Oribacterium asaccharolyticum	0,000368	0,000642	0,000647
SR1_[G-1] sp,_oral_taxon_345	0,0003264	0,00103	0,000345
Stomatobaculum sp,_oral_taxon_097	0,0004404	0,000629	0,000552
Prevotella oulorum	0,00101	0,00023	0,00029
Peptostreptococcaceae_[XI][G-5]	0,0002371	0,00099	0,000408
Solobacterium moorei	0,0004063	0,000693	0,000429
Prevotella sp,_oral_taxon_317	0,0006091	0,000427	0,000425
Bergeyella_unclassified	0,0002164	0,001212	0,000137
Capnocytophaga gingivalis	0,0006511	0,00033	0,000431
Selenomonas artemidis	0,0007026	0,000403	0,000315
Fusobacterium nucleatum_subsp,_vincentii	0,0005087	0,000731	0,000228
Bergeyella sp,_oral_taxon_931	0,0003399	0,000755	0,000351
Leptotrichia sp,_oral_taxon_417	0,0008789	0,000326	0,000177
SR1_[G-1] sp,_oral_taxon_874	0,000577	0,000279	0,000509
Pseudomonas fluorescens	0,000074	0,001227	0,000176
Lachnospiraceae_[G-2] sp,_oral_taxon_100	0,000486	0,000552	0,000311
Peptostreptococcus stomatis	0,0005373	0,000301	0,000473
Actinomyces lingnae_[NVP]	0,000393	0,000474	0,000459
Selenomonas sp,_oral_taxon_136	0,0003437	0,000441	0,000518
Leptotrichia hofstadii	0,0003131	0,000383	0,000598
Leptotrichia sp,_oral_taxon_221	0,0003473	0,000321	0,000613
Ruminococcaceae_[G-2] sp,_oral_taxon_085	0,0003263	0,000518	0,000463
Prevotella intermedia	0,0004727	0,000616	0,000228
Alloprevotella rava	0,0006131	0,000423	0,000224
Parvimonas micra	0,0004519	0,0005	0,000283
Fusobacterium sp,_oral_taxon_203	0,0003133	0,000534	0,000372
Tannerella sp,_oral_taxon_286	0,0005714	0,000252	0,000345
Porphyromonas endodontalis	0,0004956	0,000368	0,000302
Kingella sp,_oral_taxon_012	0,0004536	0,000312	0,000364

Treponema sp,_oral_taxon_237	0,0005769	0,000412	0,000146
Leptotrichia sp,_oral_taxon_212	0,0004859	0,000383	0,000232
Campylobacter sp,_oral_taxon_044	0,0002183	0,000611	0,000279
Bacteroides heparinolyticus	0,0003417	0,000367	0,000363
Prevotella nigrescens	0,0005049	8,87E-05	0,000421
Alloprevotella sp,_oral_taxon_912	0,0002849	0,00029	0,000461
Leptotrichia sp,_oral_taxon_498	5,114E-05	0,000755	0,000279
Capnocytophaga sp,_oral_taxon_338	0,0001291	0,000403	0,000484
Bacteroides sp,_oral_taxon_274	0,000355	0,000414	0,000243
Eubacterium sp,_oral_taxon_493	0,0001766	0,000514	0,000327
Kingella sp,_oral_taxon_459	0,0001916	0,000567	0,000266
Lachnoanaerobaculum orale	0,0002069	0,000281	0,000465
Actinobacteria_unclassified	0,0002846	0,000377	0,000294
Oribacterium parvum	0,0003244	0,000297	0,000311
Peptostreptococcaceae_[XI][G-7] sp,_oral_taxon_081	0,0004024	0,000288	0,000239
Neisseria oralis	0,0001444	0,000788	4,19E-05
Selenomonas sputigena	0,0002277	0,000326	0,00033
Gemella warneri	0,0003984	0,000193	0,00027
Cardiobacterium valvarum	0,0002733	0,000432	0,000163
Leptotrichia sp,_oral_taxon_215	0,0002031	0,000401	0,000243
Bacteroidetes_unclassified	0,0002541	0,000281	0,00029
Bergeyella sp,_oral_taxon_907	7,971E-05	0,000567	0,000205
Veillonellaceae_[G-1]	0,0002164	0,000321	0,000258
[Eubacterium]_yurii_subsps,_yurii_&_margaretiae	0,0004367	0,000244	8,93E-05
GN02_[G-1] sp,_oral_taxon_872	0,0004954	0,000102	0,000142
sp,_oral_taxon_458	0,000334	0,000224	0,000197
Klebsiella pneumoniae	0,000539	4,63E-05	0,00014
Neisseria bacilliformis	0,000243	4,43E-05	0,000429
Haemophilus parainfluenzae	0,000319	0,0002	0,00019
Leptotrichia wadei	0,000148	0,000403	0,000184
Bergeyella sp,_oral_taxon_319	3,714E-06	0,00076	1,13E-05
Capnocytophaga granulosa	0,0002106	0,000137	0,000336
Sneathia_unclassified	0,0001539	0,000295	0,000254
GN02_[G-1] sp,_oral_taxon_871	0,0003341	0,000361	1,31E-05
Oribacterium sinus	0,0001576	0,00012	0,000395
Mogibacterium vescum	0,0001157	0,000352	0,000234
Parvimonas alocis	0,0001557	0,00033	0,000211

Bacteroides_unclassified	0,0002166	0,00025	0,000214
Stomatobaculum longum	0,0002354	0,000166	0,000266
Corynebacterium_unclassified	0,0002336	0,000337	0,000108
Escherichia coli	0,0001633	0,000157	0,000323
Bacteroidetes_[G-5] sp,_oral_taxon_511	0,0001556	0,000295	0,000211
Fretibacterium sp,_oral_taxon_361	0,0001613	0,000208	0,00027
Capnocytophaga sp,_oral_taxon_336	0,000226	0,000264	0,000148
Veillonellaceae_[G-1] sp,_oral_taxon_155	0,0001783	0,000259	0,000184
Treponema sp,_oral_taxon_951	0,000393	0,000173	3,41E-05
[Eubacterium]_saphenum	6,071E-05	0,000476	8,17E-05
Fretibacterium fastidiosum	0,0001081	0,000166	0,000287
Leptotrichia goodfellowii	0,0001747	0,000279	0,000112
Lachnospiraceae_[G-2]_unclassified	0,0001744	0,000292	9,11E-05
Dialister pneumosintes	0,0001803	0,000293	8,54E-05
Prevotella maculosa	0,0002544	0,000139	0,000135
Prevotella saccharolytica	0,0001594	0,000255	0,000123
Peptococcus sp,_oral_taxon_167	0,0002181	0,000142	0,000161
Prevotella sp,_oral_taxon_475	0,0001443	0,00014	0,000228
[Eubacterium] sp,_oral_taxon_091	0,0002847	0,000162	6,27E-05
Actinomycetales_unclassified	0,0001177	0,000303	9,49E-05
Prevotella sp,_oral_taxon_300	8,914E-05	0,000228	0,000186
Lachnospiraceae_[G-2] sp,_oral_taxon_500	0,0002297	7,75E-05	0,000175
Prevotella aurantiaca	0,0003454	7,53E-05	4,17E-05
Simonsiella muelleri	0,0001441	0,000233	0,000108
Parvimonas_unclassified	0,0001633	0,000255	6,64E-05
Selenomonas sp,_oral_taxon_126	0,0001936	9,53E-05	0,000154
Campylobacter concisus	0,0001044	0,000093	0,000245
Actinobacteria sp,_oral_taxon_183	0,0001443	0,000122	0,000171
Leptotrichia sp,_oral_taxon_218	0,000093	0,000179	0,000173
Tannerella forsythia	0,000262	9,97E-05	6,26E-05
Catonella sp,_oral_taxon_166	0,0001463	0,00021	7,97E-05
Streptococcus anginosus	1,514E-05	0,000177	0,000232
SR1_[G-1]_unclassified	0,000076	0,000255	0,000104
Streptococcus mutans	3,714E-06	2,65E-05	0,000368
Leptotrichia sp,_oral_taxon_223	0,0001613	0,000175	8,16E-05
Atopobium parvulum	0,000152	7,28E-05	0,000175
Oribacterium sp,_oral_taxon_078	5,714E-05	0,000073	0,000268

Granulicatella_unclassified	8,743E-05	0,00029	4,54E-05
Capnocytophaga sp,_oral_taxon_326	0,0001689	0,000162	7,21E-05
Treponema socranskii	0,0001709	0,00016	5,89E-05
Capnocytophaga haemolytica	0,0002183	5,75E-05	9,13E-05
Treponema sp,_oral_taxon_246	0,0002563	0,000111	7,57E-06
Prevotella sp,_oral_taxon_443	0,000131	0,000146	0,000091
Catonella ignava	0,0001689	0,000157	4,39E-05
Leptotrichia shahii	6,071E-05	0,000162	0,000146
Veillonella rogosae	0,0001099	0,000135	0,000108
Prevotella pleuritidis	0,0001349	0,000126	7,79E-05
Mycoplasma faucium	6,271E-05	0,00023	5,11E-05
Prevotella fusca	0,000112	0,000042	0,000161
Tannerella_unclassified	0,0002106	3,32E-05	6,84E-05
Peptostreptococcaceae_[XI][G-9]	0,0001159	0,000133	7,59E-05
[Eubacterium]_brachy	0,0001159	0,000133	7,59E-05
Peptostreptococcaceae_[XI][G-7]_unclassified	6,457E-05	0,000193	6,64E-05
Fretibacterium_unclassified	0,000074	0,000111	0,000127
Leptotrichia sp,_oral_taxon_225	0,0001251	0,000122	5,89E-05
Bacilli_unclassified	7,029E-05	0,000184	5,31E-05
Lachnoanaerobaculum_unclassified	8,143E-05	0,000122	7,96E-05
Peptostreptococcaceae_[XI][G-6]	3,786E-05	0,000235	2,66E-05
Porphyromonas uenonis	4,371E-05	0,000184	6,07E-05
Lachnospiraceae_[G-2] sp,_oral_taxon_088	0,0001329	0,000144	1,86E-06
Comamonadaceae_unclassified	3,243E-05	0,000182	6,27E-05
Comamonadaceae_unclassified	3,243E-05	0,000182	6,27E-05
Proteobacteria_unclassified	8,357E-05	0,000126	5,69E-05
Proteobacteria_unclassified	8,357E-05	0,000126	5,69E-05
Rothia_unclassified	0,0001004	6,22E-05	8,91E-05
Lachnoanaerobaculum saburreum	6,257E-05	0,000164	3,99E-05
Anaeroglobus geminatus	5,514E-05	0,000151	5,69E-05
Bacteroidetes_[G-5] sp,_oral_taxon_505	0,0001386	9,97E-05	1,51E-05
Lachnospiraceae_[G-2] sp,_oral_taxon_096	9,314E-05	0,000124	3,99E-05
Streptococcus sp,_oral_taxon_093	3,229E-05	0,000157	6,84E-05
[Eubacterium]_nodatum	0,000036	0,000204	2,47E-05
Stenotrophomonas	0	4,22E-05	0,000196
Stenotrophomonas maltophilia	0	4,22E-05	0,000196
Gemella_unclassified	0,000074	0,000122	4,74E-05

Lactobacillus_unclassified	2,457E-05	0,000208	2,27E-05
Mitsuokella sp,_oral_taxon_521	4,371E-05	0,000051	0,000139
Catonella sp,_oral_taxon_451	4,929E-05	9,75E-05	0,000091
Leptotrichia sp,_oral_taxon_219	7,214E-05	0,000124	4,56E-05
Bifidobacterium wiggsiae	0,0001234	1,33E-05	8,34E-05
Acinetobacter sp,_oral_taxon_408	1,329E-05	0,000228	0
Treponema sp,_oral_taxon_250	0,0001614	4,87E-05	5,71E-06
Campylobacter gracilis	0,0001424	0,000031	0,000038
Prevotella dentalis	0,000072	0,000109	0,000038
Acinetobacter_unclassified	0,0001956	0,000009	0
GN02 sp,_oral_taxon_873	0,000038	0,000164	0,000021
Treponema denticola	3,971E-05	0,000155	2,47E-05
Butyrivibrio sp,_oral_taxon_080	1,714E-05	1,55E-05	0,000165
Mycoplasma salivarium	0,0001157	3,77E-05	4,37E-05
Pedobacter_unclassified	0	0,000219	0
Treponema lecithinolyticum	0,00011	5,53E-05	3,03E-05
Prevotella micans	0,000095	8,63E-05	1,51E-05
Acinetobacter baumannii	0,0001729	8,83E-06	1,86E-06
Prevotella sp,_oral_taxon_396	4,371E-05	8,88E-05	6,09E-05
Ottowia sp,_oral_taxon_894	5,129E-05	0,000106	3,79E-05
Capnocytophaga sp,_oral_taxon_412	5,514E-05	3,55E-05	8,73E-05
Prevotella sp,_oral_taxon_315	5,514E-05	8,18E-05	3,97E-05
Achromobacter xylosoxidans	0	0	0,00015
Prevotella baroniae	0,000074	5,77E-05	2,46E-05
Actinomyces gerencseriae	2,271E-05	1,98E-05	0,000104
Atopobium sp,_oral_taxon_199	1,329E-05	3,12E-05	0,000104
Actinomyces sp,_oral_taxon_448	5,886E-05	4,87E-05	0,000036
Tannerella sp,_oral_taxon_916	0,0001177	1,55E-05	5,71E-06
Lactobacillusfermentum	0	0,000042	0,000101
Treponema sp,_oral_taxon_263	0,000036	0,000062	4,56E-05
Streptococcus sp,_oral_taxon_402	2,471E-05	0,00006	5,69E-05
Veillonellaceae_[G-1] sp,_oral_taxon_150	2,857E-05	0,000053	5,13E-05
Cardiobacterium_unclassified	3,614E-05	0,000082	0,000019
Bacteroidetes_[G-3] sp,_oral_taxon_503	1,514E-05	0,000062	5,11E-05
Desulfovibrio fairfieldensis	0,000019	4,5E-06	9,69E-05
Actinobacteria sp,_oral_taxon_848	3,414E-05	4,87E-05	3,99E-05
Bacteroidetes_[G-5] sp,_oral_taxon_507	2,271E-05	0,000106	0

Lactobacillus gasseri	1,143E-05	1,55E-05	8,73E-05
Lachnoanaerobaculum sp,_oral_taxon_496	0	2,88E-05	8,73E-05
Oribacterium satelles	3,786E-05	2,87E-05	4,74E-05
Selenomonas dianae	5,714E-06	1,98E-05	8,56E-05
Prevotella sp,_oral_taxon_305	3,614E-05	4,43E-05	3,23E-05
Prevotella sp,_oral_taxon_942	6,257E-05	4,5E-06	3,99E-05
Delftia acidovorans	1,857E-06	1,77E-05	8,73E-05
Sphingomonas echinoides	3,857E-06	0,000113	0
Rothia omnicolens	4,557E-05	2,17E-06	5,13E-05
Dialister_unclassified	4,357E-05	2,65E-05	3,01E-05
Betaproteobacteria_unclassified	4,557E-05	2,43E-05	2,84E-05
Betaproteobacteria_unclassified	4,557E-05	2,43E-05	2,84E-05
Selenomonas flueggei	1,329E-05	1,12E-05	7,01E-05
Mogibacterium timidum	3,857E-06	9,73E-05	3,71E-06
Erysipelotrichaceae_[G-1] sp,_oral_taxon_905	9,571E-06	0,000011	7,21E-05
Moraxella catarrhalis	7,214E-05	1,77E-05	1,86E-06
Bifidobacterium denticolens	9,429E-06	8,87E-05	0
Treponema pectinovorum	0	8,83E-06	7,79E-05
Bacteroidetes_[G-3] sp,_oral_taxon_280	2,086E-05	6,87E-05	3,71E-06
Moraxella osloensis	6,071E-05	1,98E-05	1,86E-06
Treponema sp,_oral_taxon_227	7,571E-06	6,65E-05	1,14E-05
Treponema maltophilum	2,657E-05	0,000031	2,09E-05
Prevotella sp,_oral_taxon_526	2,271E-05	2,65E-05	2,66E-05
Treponema sp,_oral_taxon_257	1,514E-05	6,67E-06	5,13E-05
Haemophilus sp,_oral_taxon_036	2,086E-05	0,000031	2,29E-05
Moraxella_unclassified	1,329E-05	5,53E-05	5,71E-06
Treponema sp,_oral_taxon_927	5,714E-06	2,43E-05	3,99E-05
Bifidobacterium dentium	5,714E-06	6,67E-06	5,13E-05
Prevotella buccae	3,229E-05	2,22E-05	1,14E-05
Prevotella marshii	3,043E-05	2,22E-05	1,33E-05
Veillonellaceae_unclassified	9,429E-06	3,32E-05	2,26E-05
Veillonellaceae_unclassified	9,429E-06	3,32E-05	2,26E-05
Treponema sp,_oral_taxon_234	3,043E-05	2,65E-05	5,71E-06
Dialister micraerophilus	7,571E-06	0	4,93E-05
Prevotella shahii	1,143E-05	2,22E-05	2,49E-05
Bifidobacterium_unclassified	2,471E-05	8,83E-06	2,09E-05
Mycoplasma orale	1,529E-05	1,33E-05	2,66E-05

Burkholderiaceae_unclassified	1,343E-05	2,67E-05	0,000017
Burkholderiaceae_unclassified	1,343E-05	2,67E-05	0,000017
Gammaproteobacteria_unclassified	4,171E-05	1,33E-05	0
Gammaproteobacteria_unclassified	4,171E-05	1,33E-05	0
Prevotella enoeca	3,043E-05	1,33E-05	9,57E-06
GN02_[G-1]_unclassified	0,000036	1,77E-05	0
Treponema sp,_oral_taxon_256	1,143E-05	0,000011	3,04E-05
Treponema sp,_oral_taxon_226	3,229E-05	1,55E-05	1,86E-06
Treponema parvum	2,271E-05	8,83E-06	1,51E-05
Treponema sp,_oral_taxon_490	1,329E-05	2,88E-05	7,57E-06
Prevotella sp,_oral_taxon_313	3,029E-05	8,83E-06	5,71E-06
Granulicatella faecalis	0	0,000051	0
Olsenella sp,_oral_taxon_807	3,714E-06	2,65E-05	1,51E-05
Bacteroidetes_[G-3]_unclassified	2,843E-05	1,55E-05	0
Prevotella oralis	9,429E-06	0,00002	1,51E-05
Erysipelotrichaceae_[G-1] sp,_oral_taxon_904	9,429E-06	1,55E-05	0,000019
[Eubacterium]_infirmum	2,471E-05	8,83E-06	7,57E-06
Butyrivibrio_unclassified	1,329E-05	1,77E-05	9,43E-06
Mycoplasma_unclassified	5,714E-06	2,43E-05	1,14E-05
Bacteroidetes sp,_oral_taxon_272	7,571E-06	2,88E-05	3,86E-06
Treponema sp,_oral_taxon_508	5,714E-06	2,43E-05	9,43E-06
Actinomyces dentalis	1,329E-05	1,32E-05	9,43E-06
Peptostreptococcaceae_[XI][G-4]	1,143E-05	2,42E-05	1,86E-06
Peptostreptococcaceae_[XI][G-4]_unclassified	1,143E-05	2,42E-05	1,86E-06
Leptotrichiaceae_unclassified	1,329E-05	8,83E-06	1,33E-05
Leptotrichiaceae_unclassified	1,329E-05	8,83E-06	1,33E-05
Pseudomonas_unclassified	3,229E-05	0	1,86E-06
Atopobium exigua	5,714E-06	0	2,47E-05
Peptostreptococcaceae_[XI][G-6]_unclassified	1,857E-06	0,000031	1,86E-06
Haemophilus influenzae	9,429E-06	8,83E-06	1,31E-05
Pseudomonas aeruginosa	0	0,00002	1,33E-05
Actinomyces sp,_oral_taxon_896	9,571E-06	2,17E-06	1,71E-05
Bacteroides pyogenes	1,143E-05	1,33E-05	5,71E-06
Streptococcus agalactiae	2,471E-05	0	3,86E-06
Lachnospiraceae_[G-2] sp,_oral_taxon_086	5,571E-06	1,77E-05	7,57E-06
Megasphaera sp,_oral_taxon_123	3,857E-06	8,83E-06	1,71E-05
