# Supplementary material for: A 7-Week Summer Camp in Antarctica Induces Fluctuations on Human Oral Microbiome, Pro-Inflammatory Markers and Metabolic Hormones Profile
Source: Microorganisms. 2023 Jan 30;11(2):339. doi: 10.3390/microorganisms11020339 (PMC9960157; doi:10.3390/microorganisms11020339)
Supplement: Supplementary file 1 [file microorganisms-11-00339-s001.zip › _.Suppl Material 5. Corrected Proof.pdf]

Association between the relative abundance of bacterial genus and diversity indexes with cytokine and signal proteins

Michele M Moraes, Thiago T Mendes, Leandro Borges, Alice L Marques, Cristian Núñez-Espinosa, Dawit AP Gonçalves, Carolina B Simões, Tales S Vieira, Roberto VP Ladeira, Talita GB Lourenco, Danielle V Ribeiro, Elaine Hatanaka, Debora Heller, Rosa ME Arantes

This is Supplemental material S5 for the article: **A 7-week summer camp in Antarctica induces fluctuations on human oral microbiome, pro-inflammatory markers and metabolic hormones profile**

Table S5. Results of Spearman's correlations between the relative abundance of bacterial genus and diversity indexes with cytokine and signal proteins.

	Genus	Leptin	IL-8	IL1-β	IL-6	VEGF	hs-CRP	TNF-α
1	**Clostridiales_unclassified**	r = 0.332<br>p = 0.149	r = -0.013<br>p = 0.952	r = -0.313<br>p = 0.176	r = 0.029<br>p = 0.901	r = -0.44<br>p = 0.058	r = -0.225<br>p = 0.333	r = -0.107<br>p = 0.657
2	**Granulicatella**	r = 0.402<br>p = 0.078	r = -0.383<br>p = 0.093	r = **-0.704** *<br>p = 0.001	r = 0.146<br>p = 0.534	r = **-0.593** *<br>p = 0.007	r = -0.341<br>p = 0.138	r = **-0.482** *<br>p = 0.036
3	**Mogibacterium**	r = -0.105<br>p = 0.662	r = **-0.452** *<br>p = 0.050	r = -0.415<br>p = 0.075	r = -0.186<br>p = 0.439	r = -0.198<br>p = 0.421	r = -0.334<br>p = 0.157	r = **-0.533** *<br>p = 0.022
4	**Firmicutes_unclassified**	r = 0.318<br>p = 0.169	r = -0.013<br>p = 0.952	r = -0.263<br>p = 0.257	r = 0.079<br>p = 0.733	r = -0.265<br>p = 0.268	r = -0.147<br>p = 0.529	r = -0.147<br>p = 0.541
5	**Ruminococcaceae_[G-1]**	r = -0.037<br>p = 0.871	r = -0.156<br>p = 0.504	r = 0.044<br>p = 0.846	r = 0.212<br>p = 0.364	r = 0.055<br>p = 0.815	r = 0.070<br>p = 0.762	r = -0.149<br>p = 0.536
6	**Veillonellaceae_[G-1]**	r = -0.258<br>p = 0.293	r = -0.116<br>p = 0.638	r = 0.103<br>p = 0.674	r = -0.093<br>p = 0.705	r = 0.063<br>p = 0.802	r = -0.131<br>p = 0.597	r = -0.308<br>p = 0.222
7	**Abiotrophia**	r = **0.489** *<br>p = 0.029	r = -0.317<br>p = 0.169	r = -0.278<br>p = 0.230	r = **0.423** *<br>p = 0.062	r = -0.181<br>p = 0.453	r = 0.015<br>p = 0.947	r = -0.046<br>p = 0.848
8	**Lachnoanaerobaculum**	r = 0.212<br>p = 0.377	r = 0.028<br>p = 0.905	r = -0.040<br>p = 0.865	r = 0.107<br>p = 0.657	r = 0.059<br>p = 0.811	r = -0.226<br>p = 0.345	r = 0.020<br>p = 0.934
9	**Solobacterium**	r = 0.181<br>p = 0.453	r = 0.103<br>p = 0.667	r = -0.015<br>p = 0.945	r = 0.214<br>p = 0.373	r = 0.038<br>p = 0.876	r = -0.129<br>p = 0.590	r = 0.041<br>p = 0.863
10	**Gemella**	r = **0.486** *<br>p = 0.030	r = -0.039<br>p = 0.866	r = -0.278<br>p = 0.230	r = 0.514<br>p = 0.02	r = -0.219<br>p = 0.361	r = **-0.442** *<br>p = 0.050	r = -0.126<br>p = 0.600
11	**Filifactor**	r = **-0.488** *<br>p = 0.039	r = 0.031<br>p = 0.895	r = 0.238<br>p = 0.334	r = -0.34<br>p = 0.164	r = 0.402<br>p = 0.107	r = 0.066<br>p = 0.792	r = -0.206<br>p = 0.420
12	**Oribacterium**	r = 0.020<br>p = 0.932	r = 0.093<br>p = 0.691	r = 0.080<br>p = 0.733	r = 0.078<br>p = 0.738	r = -0.116<br>p = 0.631	r = -0.143<br>p = 0.542	r = -0.319<br>p = 0.179
13	**Streptococcus**	r = **0.412** *<br>p = 0.070	r = -0.098<br>p = 0.676	r = **-0.475** *<br>p = 0.034	r = 0.182<br>p = 0.437	r = **-0.467** *<br>p = 0.043	r = **-0.568** *<br>p = 0.009	r = -0.205<br>p = 0.393
14	**Ruminococcaceae_[G-2]**	r = 0.025<br>p = 0.911	r = **0.449** *<br>p = 0.046	r = **0.454** *<br>p = 0.044	r = 0.226<br>p = 0.330	r = 0.374<br>p = 0.112	r = 0.323<br>p = 0.161	r = 0.207<br>p = 0.389
15	**Lachnospiraceae_[XIV]_unclassified**	r = -0.005<br>p = 0.982	r = 0.023<br>p = 0.921	r = -0.036<br>p = 0.876	r = 0.048<br>p = 0.836	r = 0.002<br>p = 0.991	r = 0.084<br>p = 0.719	r = -0.332<br>p = 0.162
16	**Peptostreptococcaceae_[XI][G-5]**	r = -0.164<br>p = 0.498	r = -0.049<br>p = 0.837	r = 0.135<br>p = 0.575	r = 0.040<br>p = 0.865	r = 0.107<br>p = 0.668	r = -0.077<br>p = 0.748	r = -0.189<br>p = 0.446
17	**Lactobacillus**	r = 0.381<br>p = 0.095	r = 0.092<br>p = 0.695	r = -0.305<br>p = 0.187	r = 0.143<br>p = 0.542	r = -0.317<br>p = 0.184	r = -0.344<br>p = 0.134	r = -0.246<br>p = 0.305
18	**Veillonella**	r = 0.302<br>p = 0.191	r = 0.277<br>p = 0.233	r = 0.129<br>p = 0.581	r = 0.286<br>p = 0.218	r = 0.279<br>p = 0.242	r = -0.075<br>p = 0.748	r = 0.333<br>p = 0.159
19	**Dialister**	r = -0.095<br>p = 0.686	r = 0.382<br>p = 0.095	r = 0.355<br>p = 0.122	r = 0.144<br>p = 0.538	r = 0.405<br>p = 0.0833	r = -0.102<br>p = 0.663	r = 0.211<br>p = 0.381
20	**Catonella**	r = 0.302<br>p = 0.191	r = -0.143<br>p = 0.542	r = 0.015<br>p = 0.947	r = 0.438<br>p = 0.053	r = -0.009<br>p = 0.968	r = 0.102<br>p = 0.663	r = -0.021<br>p = 0.928
21	**SR1_[G-1]**	r = -0.080<br>p = 0.733	r = 0.048<br>p = 0.836	r = 0.195<br>p = 0.403	r = 0.121<br>p = 0.603	r = 0.273<br>p = 0.252	r = 0.177<br>p = 0.448	r = 0.060<br>p = 0.798
22	**Sneathia**	r = -0.054<br>p = 0.820	r = **-0.603** *<br>p = 0.006	r = **-0.549** *<br>p = 0.015	r = -0.30<br>p = 0.207	r = -0.178<br>p = 0.471	r = -0.057<br>p = 0.809	r = -0.275<br>p = 0.263
23	**Leptotrichia**	r = 0.253<br>p = 0.277	r = 0.161<br>p = 0.492	r = 0.155<br>p = 0.508	r = 0.317<br>p = 0.170	r = 0.039<br>p = 0.871	r = 0.293<br>p = 0.205	r = -0.188<br>p = 0.435
24	**Fusobacterium**	r = -0.020<br>p = 0.927	r = -0.014<br>p = 0.947	r = 0.105<br>p = 0.653	r = 0.171<br>p = 0.464	r = 0.019<br>p = 0.934	r = 0.352<br>p = 0.125	r = -0.168<br>p = 0.484
25	**Bergeyella**	r = 0.007<br>p = 0.972	r = **-0.589** *<br>p = 0.006	r = -0.412<br>p = 0.070	r = 0.120<br>p = 0.608	r = -0.254<br>p = 0.288	r = -0.138<br>p = 0.555	r = -0.372<br>p = 0.114
26	**Alloprevotella**	r = -0.016<br>p = 0.942	r = 0.086<br>p = 0.714	r = 0.239<br>p = 0.305	r = 0.179<br>p = 0.444	r = 0.270<br>p = 0.258	r = 0.131<br>p = 0.577	r = 0.068<br>p = 0.776

**27**	**Bacteroidetes_unclassified**	r = 0.192<br>p = 0.410	r = -0.055<br>p = 0.811	r = **-0.451** *<br>p = 0.045	r = -0.158<br>p = 0.500	r = -0.368<br>p = 0.118	r = -0.125<br>p = 0.594	r = **-0.456** *<br>p = 0.049
**28**	**Capnocytophaga**	r = 0.126<br>p = 0.590	r = -0.11<br>p = 0.639	r = -0.174<br>p = 0.456	r = 0.087<br>p = 0.710	r = 0.068<br>p = 0.776	r = 0.247<br>p = 0.289	r = 0.023<br>p = 0.922
**29**	**Prevotella**	r = 0.078<br>p = 0.738	r = 0.298<br>p = 0.198	r = 0.352<br>p = 0.125	r = 0.298<br>p = 0.198	r = 0.242<br>p = 0.312	r = 0.092<br>p = 0.695	r = 0.046<br>p = 0.848
**30**	**Bacteroides**	r = 0.168<br>p = 0.472	r = 0.087<br>p = 0.710	r = -0.088<br>p = 0.705	r = -0.035<br>p = 0.881	r = -0.246<br>p = 0.305	r = 0.286<br>p = 0.218	r = -0.136<br>p = 0.570
**31**	**Bacteroidales_unclassified**	r = 0.024<br>p = 0.916	r = -0.360<br>p = 0.116	r = **-0.599** *<br>p = 0.005	r = -0.289<br>p = 0.213	r = -0.562<br>p = 0.012	r = -0.097<br>p = 0.676	r = **-0.658** *<br>p = 0.002
**32**	**Actinomyces**	r = 0.122<br>p = 0.603	r = -0.159<br>p = 0.496	r = -0.305<br>p = 0.187	r = 0.134<br>p = 0.568	r = -0.375<br>p = 0.111	r = -0.066<br>p = 0.777	r = -0.404<br>p = 0.085
**33**	**Corynebacterium**	r = -0.095<br>p = 0.686	r = -0.037<br>p = 0.871	r = -0.15<br>p = 0.521	r = -0.205<br>p = 0.378	r = -0.125<br>p = 0.600	r = -0.396<br>p = 0.081	r = -0.248<br>p = 0.298
**34**	**Actinobacteria_unclassified**	r = -0.093<br>p = 0.691	r = -0.008<br>p = 0.967	r = 0.127<br>p = 0.585	r = 0.173<br>p = 0.460	r = 0.037<br>p = 0.877	r = 0.166<br>p = 0.480	r = -0.091<br>p = 0.705
**35**	**Rothia**	r = 0.047<br>p = 0.841	r = 0.044<br>p = 0.846	r = -0.252<br>p = 0.277	r = -0.090<br>p = 0.700	r = **-0.477** *<br>p = 0.038	r = **-0.505** *<br>p = 0.023	r = **-0.628** *<br>p = 0.004
**36**	**Kingella**	r = 0.244<br>p = 0.295	r = -0.227<br>p = 0.330	r = -0.235<br>p = 0.314	r = 0.089<br>p = 0.700	r = -0.161<br>p = 0.503	r = 0.39<br>p = 0.088	r = -0.042<br>p = 0.860
**37**	**Eikenella**	r = 0.290<br>p = 0.224	r = 0.106<br>p = 0.657	r = -0.122<br>p = 0.610	r = 0.184<br>p = 0.444	r = 0.002<br>p = 0.987	r = 0.161<br>p = 0.503	r = -0.156<br>p = 0.530
**38**	**Neisseriaceae_unclassified**	r = 0.149<br>p = 0.525	r = 0.178<br>p = 0.444	r = 0.265<br>p = 0.255	r = 0.387<br>p = 0.090	r = 0.379<br>p = 0.107	r = 0.371<br>p = 0.105	r = 0.267<br>p = 0.264
**39**	**Neisseria**	r = -0.026<br>p = 0.911	r = 0.105<br>p = 0.653	r = 0.310<br>p = 0.180	r = 0.274<br>p = 0.238	r = 0.377<br>p = 0.109	r = 0.280<br>p = 0.228	r = 0.356<br>p = 0.131
**40**	**Escherichia**	r = -0.084<br>p = 0.736	r = 0.360<br>p = 0.138	r = 0.184<br>p = 0.456	r = -0.206<br>p = 0.407	r = -0.117<br>p = 0.646	r = **0.474** *<br>p = 0.046	r = -0.262<br>p = 0.302
**41**	**Stenotrophomonas**	r = 0.372<br>p = 0.137	r = -0.369<br>p = 0.142	r = **-0.629** *<br>p = 0.007	r = 0.135<br>p = 0.598	r = **-0.659** *<br>p = 0.005	r = **-0.527** *<br>p = 0.029	r = **-0.556** *<br>p = 0.0247
**42**	**Treponema**	r = 0.019<br>p = 0.932	r = -0.197<br>p = 0.399	r = -0.070<br>p = 0.762	r = 0.173<br>p = 0.460	r = -0.025<br>p = 0.917	r = 0.044<br>p = 0.851	r = -0.311<br>p = 0.191
**43**	**Fretibacterium**	r = 0.0321<br>p = 0.894	r = -0.110<br>p = 0.646	r = 0.062<br>p = 0.798	r = 0.267<br>p = 0.264	r = -0.039<br>p = 0.876	r = -0.205<br>p = 0.393	r = -0.248<br>p = 0.313
**44**	**Porphyromonas**	r = 0.137<br>p = 0.559	r = 0.102<br>p = 0.663	r = 0.180<br>p = 0.440	r = 0.284<br>p = 0.220	r = 0.272<br>p = 0.255	r = 0.367<br>p = 0.109	r = -0.026<br>p = 0.911
**45**	**Bacteroidales_[G-2]**	r = -0.040<br>p = 0.865	r = 0.040<br>p = 0.865	r = 0.214<br>p = 0.373	r = 0.107<br>p = 0.657	r = 0.267<br>p = 0.278	r = 0.168<br>p = 0.484	r = 0.044<br>p = 0.856
**46**	**Bacteroidetes_[G-5]**	r = 0.110<br>p = 0.646	r = -0.102<br>p = 0.673	r = -0.026<br>p = 0.911	r = 0.309<br>p = 0.194	r = 0.103<br>p = 0.674	r = -0.067<br>p = 0.781	r = -0.170<br>p = 0.492
**47**	**Tannerella**	r = 0.149<br>p = 0.525	r = 0.259<br>p = 0.266	r = 0.294<br>p = 0.203	r = 0.296<br>p = 0.201	r = 0.333<br>p = 0.159	r = 0.143<br>p = 0.542	r = 0.061<br>p = 0.790
**48**	**Megasphaera**	r = 0.033<br>p = 0.886	r = 0.344<br>p = 0.134	r = 0.256<br>p = 0.269	r = 0.325<br>p = 0.159	r = 0.143<br>p = 0.550	r = -0.141<br>p = 0.546	r = 0.128<br>p = 0.595
**49**	**Peptostreptococcaceae_[XI] [G-7]**	r = 0.055<br>p = 0.811	r = **-0.451** *<br>p = 0.045	r = -0.303<br>p = 0.191	r = 0.190<br>p = 0.414	r = -0.076<br>p = 0.748	r = -0.211<br>p = 0.364	r = -0.372<br>p = 0.114
**50**	**Stomatobaculum**	r = -0.003<br>p = 0.986	r = 0.032<br>p = 0.894	r = 0.226<br>p = 0.345	r = 0.165<br>p = 0.493	r = 0.441<br>p = 0.066	r = -0.074<br>p = 0.759	r = 0.271<br>p = 0.270
**51**	**Selenomonas**	r = 0.203<br>p = 0.385	r = 0.197<br>p = 0.399	r = 0.0391<br>p = 0.866	r = 0.298<br>p = 0.198	r = 0.023<br>p = 0.922	r = -0.17<br>p = 0.468	r = -0.270<br>p = 0.258
**52**	**Peptostreptococcus**	r = -0.295<br>p = 0.203	r = -0.168<br>p = 0.472	r = -0.075<br>p = 0.748	r = -0.317<br>p = 0.169	r = 0.275<br>p = 0.249	r = -0.054<br>p = 0.816	r = 0.170<br>p = 0.480
**53**	**Lachnospiraceae_[G-3]**	r = 0.211<br>p = 0.367	r = -0.403<br>p = 0.077	r = -0.397<br>p = 0.081	r = 0.145<br>p = 0.534	r = -0.071<br>p = 0.770	r = -0.085<br>p = 0.719	r = -0.264<br>p = 0.268
**54**	**Peptostreptococcaceae_[XI] [G-1]**	r = -0.234<br>p = 0.327	r = 0.046<br>p = 0.848	r = 0.339<br>p = 0.153	r = -0.005<br>p = 0.980	r = 0.393<br>p = 0.104	r = 0.102<br>p = 0.673	r = -0.018<br>p = 0.941
**55**	**Parvimonas**	r = 0.009<br>p = 0.967	r = -0.335<br>p = 0.145	r = -0.305<br>p = 0.187	r = 0.020<br>p = 0.932	r = -0.146<br>p = 0.546	r = -0.295<br>p = 0.203	r = **-0.474** *<br>p = 0.040
**56**	**Staphylococcus**	r = -0.078<br>p = 0.738	r = 0.385<br>p = 0.092	r = **0.474** *<br>p = 0.035	r = 0.262<br>p = 0.260	r = 0.439<br>p = 0.059	r = 0.069<br>p = 0.767	r = 0.429<br>p = 0.065
**57**	**Atopobium**	r = 0.109<br>p = 0.652	r = -0.054<br>p = 0.820	r = -0.135<br>p = 0.575	r = 0.118<br>p = 0.626	r = 0.013<br>p = 0.954	r = -0.085<br>p = 0.721	r = 0.205<br>p = 0.407
**58**	**Actinobaculum**	r = 0.397<br>p = 0.091	r = -0.037<br>p = 0.877	r = -0.224<br>p = 0.349	r = 0.193<br>p = 0.422	r = 0.066<br>p = 0.786	r = -0.187<br>p = 0.435	r = -0.213<br>p = 0.388
**59**	**Campylobacter**	r = -0.015<br>p = 0.947	r = -0.147<br>p = 0.529	r = 0.110<br>p = 0.639	r = 0.236<br>p = 0.311	r = 0.165<br>p = 0.493	r = 0.177<br>p = 0.448	r = 0.218<br>p = 0.365
**60**	**Cardiobacterium**	r = 0.409<br>p = 0.072	r = -0.129<br>p = 0.581	r = -0.107<br>p = 0.649	r = **0.481** *<br>p = 0.031	r = 0.007<br>p = 0.974	r = 0.045<br>p = 0.846	r = 0.005<br>p = 0.980

**61**	**Haemophilus**	r = -0.044<br>p = 0.851	r = 0.107<br>p = 0.649	r = 0.138<br>p = 0.555	r = -0.14<br>p = 0.551	r = 0.168<br>p = 0.484	r = 0.426<br>p = 0.0603	r = 0.249<br>p = 0.298
**62**	**Aggregatibacter**	r = 0.108<br>p = 0.644	r = 0.188<br>p = 0.421	r = 0.245<br>p = 0.292	r = 0.174<br>p = 0.456	r = 0.311<br>p = 0.191	r = 0.459<br>p = 0.0414	r = 0.121<br>p = 0.615
**63**	**Pasteurellaceae_unclassified**	r = -0.197<br>p = 0.399	r = 0.263<br>p = 0.257	r = 0.289<br>p = 0.213	r = 0.138<br>p = 0.555	r = 0.398<br>p = 0.089	r = 0.274<br>p = 0.238	r = 0.153<br>p = 0.526
**64**	**Lautropia**	r = -0.412<br>p = 0.070	r = 0.138<br>p = 0.555	r = 0.305<br>p = 0.187	r = -0.180<br>p = 0.440	r = 0.295<br>p = 0.216	r = 0.264<br>p = 0.255	r = 0.146<br>p = 0.546

Footnote: Association between the relative abundance of bacterial genus and diversity indexes with cytokine and signal proteins. IL-8: *Interleukin* 8. IL1-β*: Interleukin* 1-Beta. IL-6: *Interleukin* 6. VEGF: Vascular endothelial growth factor. hs-CRP: *high-sensitivity C-reactive protein. TNF-α: Tumor necrosis* fator-*alpha*. *Pseudomonadota*, former *Proteobacteria*. *Bacillota*, former *Firmicutes*. *Bacteroidota,* former *Bacteroidetes*. *Actinomycetota*, former *Actinobacteria*. Number of samples = 20 for all correlations, except for correlations with *TNF-α and* VEGF with a number of samples = 19, due to the limited volume of samples for one volunteer at the Final moment. Statistical analysis: Spearman Rank Order Correlation. *Significant association ($P < 0.05$) between the data.
